# Fungal and mycotoxin contaminants in cannabis and hemp flowers: implications for consumer health and directions for further research

**DOI:** 10.3389/fmicb.2023.1278189

**Published:** 2023-10-19

**Authors:** Kimberly D. Gwinn, Maxwell C. K. Leung, Ariell B. Stephens, Zamir K. Punja

**Affiliations:** ^1^Department of Entomology and Plant Pathology, University of Tennessee, Knoxville, TN, United States; ^2^School of Mathematical and Natural Sciences, Arizona State University, Glendale, AZ, United States; ^3^Department of Biological Sciences, Simon Fraser University, Burnaby, BC, Canada

**Keywords:** total yeast and mold, quality control, opportunistic infection, mycotoxins, health risks, cannabis safety

## Abstract

Medicinal and recreational uses of *Cannabis sativa*, commonly known as cannabis or hemp, has increased following its legalization in certain regions of the world. Cannabis and hemp plants interact with a community of microbes (i.e., the phytobiome), which can influence various aspects of the host plant. The fungal composition of the *C. sativa* phytobiome (i.e., mycobiome) currently consists of over 100 species of fungi, which includes phytopathogens, epiphytes, and endophytes, This mycobiome has often been understudied in research aimed at evaluating the safety of cannabis products for humans. Medical research has historically focused instead on substance use and medicinal uses of the plant. Because several components of the mycobiome are reported to produce toxic secondary metabolites (i.e., mycotoxins) that can potentially affect the health of humans and animals and initiate opportunistic infections in immunocompromised patients, there is a need to determine the potential health risks that these contaminants could pose for consumers. This review discusses the mycobiome of cannabis and hemp flowers with a focus on plant-infecting and toxigenic fungi that are most commonly found and are of potential concern (e.g., *Aspergillus, Penicillium, Fusarium,* and *Mucor* spp.). We review current regulations for molds and mycotoxins worldwide and review assessment methods including culture-based assays, liquid chromatography, immuno-based technologies, and emerging technologies for these contaminants. We also discuss approaches to reduce fungal contaminants on cannabis and hemp and identify future research needs for contaminant detection, data dissemination, and management approaches. These approaches are designed to yield safer products for all consumers.

## 1. Introduction

*Cannabis sativa* is a highly domesticated plant species that has been bred for hundreds of years to develop genotypes (i.e., strains) that are cultivated worldwide for medicinal, therapeutic, and recreational properties, as well as for grain, seed, and fiber [Krane, 2020; United States Department of Agriculture (USDA), 2020; Rafei et al., 2023]. The inflorescence tissues (which are also referred to as flowers or buds) are of significant economic value, since they can be used as dried flowers and as raw materials for cannabinoid extraction [United States Department of Agriculture-Economics, Statistics and Market Information System (USDA ESMIS), 2023]. The legal classification of genotypes depends upon content of Δ9-tetrahydrocannabinol (THC) content in the tissues, with cannabis containing levels greater than 0.3% (by dry weight) and hemp containing less than 0.3% Δ9-THC (Williams and Buchert, 2020). Cannabis and hemp can be used in a variety of ways, including as dried flowers that are smoked, inhaled, or vaped; flowers that have undergone an extraction process for cannabinoids; and formulated concentrates that include capsules, gels, creams, suppositories, and tinctures.

The mycobiome of cannabis and hemp is defined as the totality of fungal communities associated with various parts of the plant. The mycobiome is a subset of the phytobiome, which includes bacteria, phytoplasmas, viruses, and viroids that are beyond the scope of this review (Trivedi et al., 2020; Wei et al., 2021). Mycobiomes have garnered considerable recent interest within the scientific community, because they are present in virtually every plant species (Trivedi et al., 2020). Their interactions with host plants can lead to beneficial, neutral, or detrimental interactions, and conversely plant chemical composition can influence the mycobiome (Ranjith et al., 2021). Fungi that occur on the inflorescence tissues of cannabis and hemp as phytopathogens, surface entities (epiphytes) or as internal endophytes have the potential to produce toxic secondary metabolites (mycotoxins) and a few are pathogenic to humans (see Section 2 below). These fungi pose concerns for human health as they can result in opportunistic infections (i.e., mycoses) and cause toxicological effects. For the purposes of this review, the assemblage of fungi and their toxins from these sources found on cannabis and hemp are referred to as “contaminants.”

Although fungi and mycotoxins are common and well-studied contaminants in many agricultural crop species, they have been generally under-studied in cannabis and hemp. This is partly because human health risk assessment methodologies used to regulate food and pharmaceuticals have yet to become standard for the emerging cannabis and hemp industries. Additionally, the wide range of consumer uses of cannabis and hemp flowers, including for medical use by patients with susceptible conditions, makes it uniquely challenging to assess and manage human health risk of these contaminants. This review will discuss the relative importance of the floral mycobiome of cannabis and hemp emphasizing its potential to impact consumer health. We will assess the potential of cannabis and hemp contaminants to affect consumers of cannabis, discuss regulatory considerations and management practices, and highlight areas for future research.

## 2. Mycobiome of *Cannabis sativa*: phytopathogens, endophytes, and epiphytes

Fungal organisms comprising the mycobiome belong to the Kingdom Mycota and are characterized by a number of unique features, including: (i) the ability to grow rapidly on an appropriate substrate; (ii) growth over a wide range of temperatures; (iii) production of enzymes and toxins that destroy plant substrates; (iv) production of prolific numbers of asexual propagules (spores, conidia) that are easily disseminated by air and water; and (v) production of mycotoxins potentially harmful to humans and animals. The presence of fungal organisms in cannabis plants is not unique since many species comprising the floral mycobiome are also abundantly present across a wide range of agricultural crops. From the perspective of consumer health, the destruction of plant tissues and the production of spores and mycotoxins in association with cannabis and hemp plants are deemed of potential concern.

**Figure 1:**
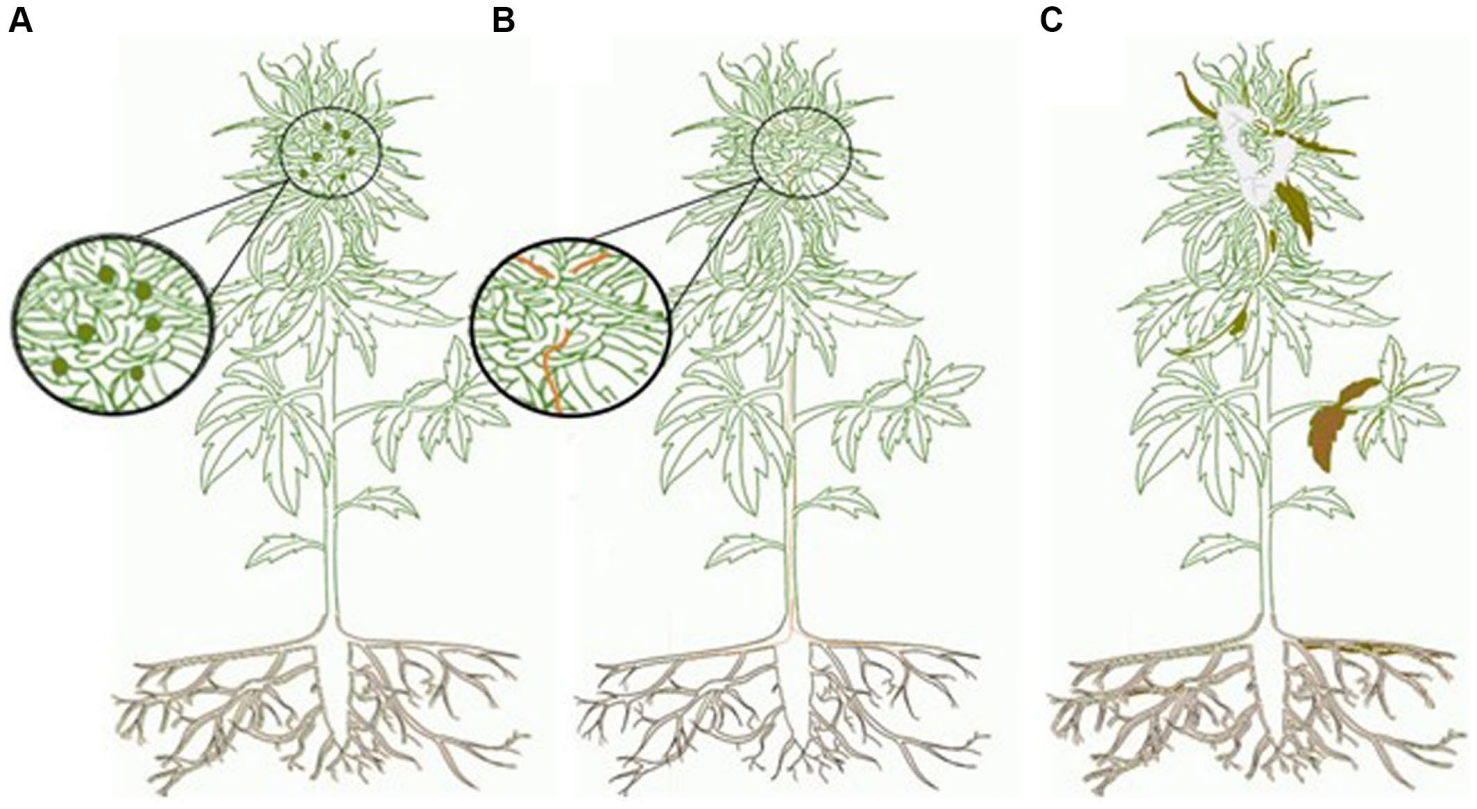
Mycobiome of cannabis and hemp. **(A)** Epiphyte—asymptomatic relationship with fungal propagules (dark green circles) that colonize only the outside of host plant. Propagules can only be observed with magnification. **(B)** Endophyte—asymptomatic relationship with fungal propagules (orange) that are found within the host. Endophytes can only be seen with dissection of the host and magnification. They may be beneficial to the plant or with an alteration in host physiology may became pathogenic. **(C)** Phytopathogen—symptomatic relationship that alters host physiology; signs (pathogen propagules—white) and symptoms (brown) may be seen without magnification. Cannabis graphic created by Maya Albert.

**Figure 2:**
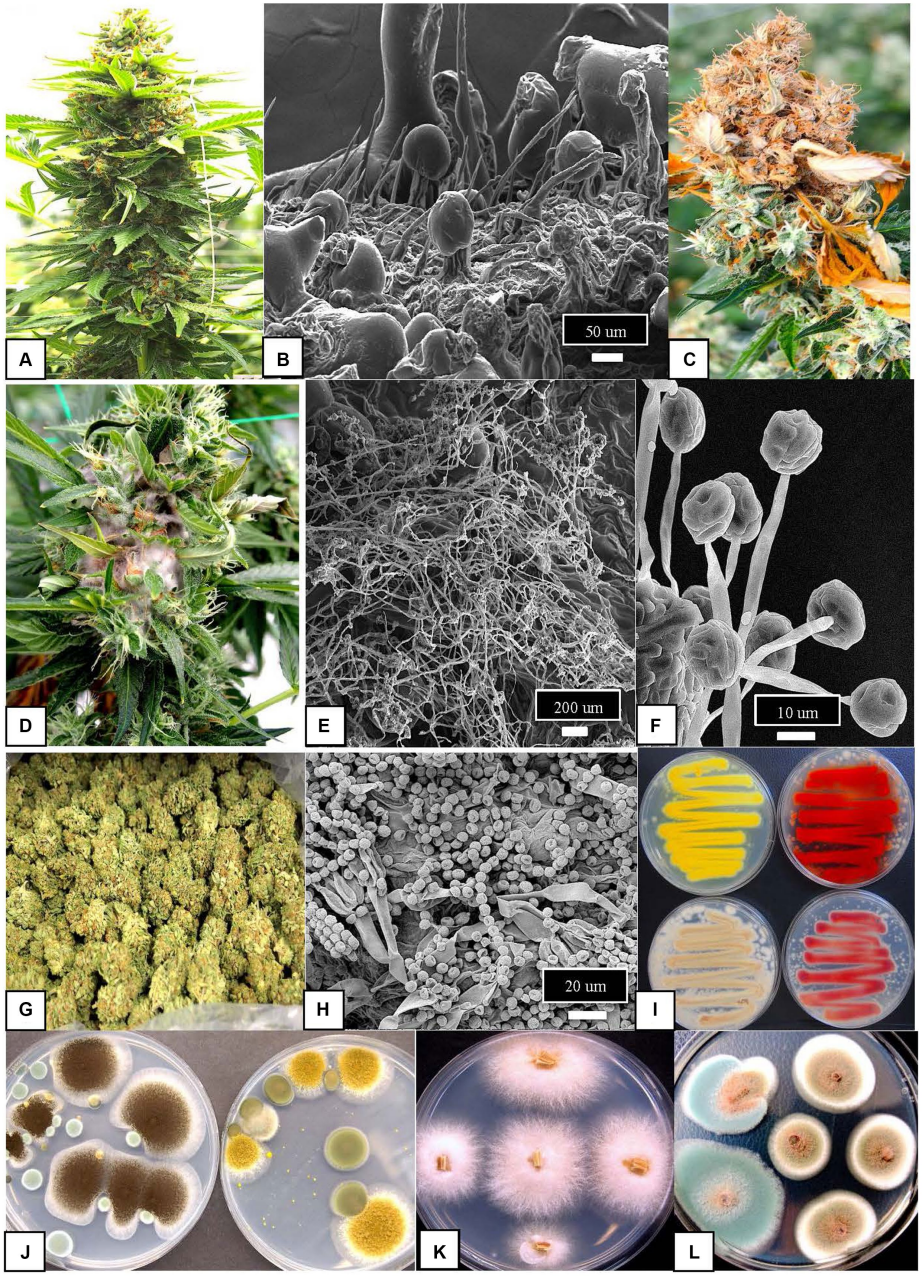
Fungal pathogens, epiphytes, and endophytes affecting cannabis inflorescences. **(A)** Cannabis inflorescence at maturation showing the large floral structure that is composed of female reproductive organs (pistils) and inflorescence leaves and bracts surrounding them. **(B)** Scanning electron micrograph of the surface of one of the bracts showing the abundance of glandular trichomes and non-glandular hairs among which fungal populations may reside. **(C)** Destruction of the inflorescence by the fungal pathogen *Botrytis cinerea* that causes browning and death of the tissues. **(D)** Proliferation of mycelium of a *Fusarium* species within the inflorescence tissues under conditions of high humidity. **(E)** Scanning electron micrograph showing growth of mycelium of a *Trichoderma* species on inflorescence tissues following a foliar application made to the plant. **(F)** Spore bearing structures of a *Gliocladium* species on inflorescence tissues following a foliar application made to the plant. In **(E,F)**, tissues were collected 5 days following application for observation. **(G)** Commercially dried cannabis inflorescences prior to packaging. **(H)** Spores of *Penicillium* species commonly observed on the surface of cannabis samples as an epiphyte either during or after the drying process. **(I)** Swabs taken from dried cannabis samples and streaked onto potato dextrose agar shows the diversity of *Penicillium* species growing on the medium and producing a range of pigments. **(J)** Growth of two *Aspergillus* species on potato dextrose agar from swabs taken from dried cannabis samples where they were present as contaminants. On the left dish is *A. niger* (black) and on the right *Aspergillus ochraceus* (yellow). **(K)** Colonies of *Fusarium oxysporum* emerging from stem pieces of cannabis plants. **(L)** Colonies of two *Penicillium* species emerging from sections of stems where they were growing as endophytes.

The cannabis and hemp mycobiomes can be subdivided into three components: phytopathogens, endophytes, and epiphytes (Figure 1). They are distinguished by where they are found on the plants and whether they cause visible damage to the plant (Figure 2). Phytopathogens cause visible disease symptoms on these plants, endophytes reside within plant tissues without causing discernable symptoms, while the epiphytes reside and grow on the outside of plant tissues and may or may not cause symptoms. According to United States National Fungus Collections and Fungal Database (NFCFD) (2023), more than 100 species of phytopathogens, epiphytes, and endophytes are associated with cannabis and hemp. The composition of this mycobiome can be influenced by host plant genotype, growth stage of the plant, external environment, and cultural practices used during commercial production (Barnett et al., 2020; Comeau et al., 2020; Punja et al., 2023). In particular, the floral mycobiome is impacted by the host genotype, host chemistry, growing environment, and pre-and post-harvest handling practices (Punja et al., 2023). Thus, the floral mycobiome of cannabis and hemp is a dynamic biological entity that is greatly influenced by plant genotype × environment interactions (Punja et al., 2023). This makes predictions and assessments of fungal population levels challenging, which can further complicate efforts to assess their potential impact on consumer health.

The floral mycobiome of cannabis and hemp plants discussed above is important for several reasons: (i) it can impact the quality of the final product; (ii) it can have an impact on consumer health; and (ii) it can pose regulatory challenges that can vary depending on the jurisdiction. Examples of fungal species associated with cannabis inflorescences and their relative abundance are summarized in Table 1. A similar study has not been conducted in hemp. In addition, the presence of airborne fungal contaminants during harvesting of cannabis and hemp and those that are present in the cultivation environment can be of potential concern for human health (Martyny et al., 2013; Vanhove et al., 2018; Root et al., 2020; Punja, 2021c; Punja and Scott, 2023). Lastly, a few fungal species of cannabis and hemp can directly impact human health by causing tissue infections, resulting in mycoses for immunocompromised individuals (see Section 3.1). For example, after inhalation of spores, these fungi can enter nasal passages and the lungs where they may cause lung infections, particularly in immunocompromised patients [National Academies of Sciences, Engineering, and Medicine (NASEM), 2017; Benedict et al., 2020].

**Table 1:** The mycobiome of cannabis inflorescences and fungal species relative abundance in samples.^a^

Genus	Species	Mycotoxin potential^b^	Relative abundance^c^
*Acremonium*	*A. alternatum*	−	<0.1%
*Alternaria*	*A. alternata*	+	10–20%
	*A. tenuissima*	+	0.1–1.0%
*Aspergillus*	*A. flavus*	+++	0.1–1.0%
	*A. niger*	+	5–10%
	*A. ochraceus*	+	30–40%
*Beauveria*	*B. bassiana*	−	0.1–1.0%
*Bjerkandera*	*B. adusta*	−	<0.1%
*Botrytis*	*B. cinerea*	+	1–5%
*Cercospora*	*C. canescents*	−	<0.1%
*Chaetomium*	*C. elatum*	+	<0.1%
	*C. brasiliensis*	+	0.1–1.0%
	*C. globosum*	+	1–5%
*Conidiobolus*	*C. coronatus*	−	0.1–1.0%
*Cladosporium*	*C. cladosporiodes (formerly C. westerdijkiae)*	+	20–30%
	*C. floccosum*	−	0.1–1.0%
*Diaporthe*	*D. eres*	_	0.1–1.0%
*Epicoccum*	*E. nigrum*	+	1–5%
*Fusarium*	*F. avenaceum*	+	<0.1%
	*F. graminearum*	+++	<0.1%
	*F. oxysporum*	+	5–10%
	*F. proliferatum*	++	0.1–1.0%
	*F. solani*	−	<0.1%
	*F. sporotrichiodes*	+++	0.1–1.0%
*Hydnopolyporus*	*H. fimbriatus*	_	0.1–1.0%
*Lasiodiplodia*	*L. theobromae*	+	0.1–1.0%
*Lecanocillium*	*L. aphanocladii*	−	0.1–1.0%
*Metarhizium*	*M. anisopliae*	−	0.1–1.0%
*Mortierella*	*M. hyaline*	_	5–10%
*Mucor*	*M. circinelloides*	+	10–20%
	*M. racemosus*	+	10–20%
*Nigrospora*	*N. oryzae*	−	0.1–1.0%
*Paraphaeosphaeria*	*P. michotii*	_	<0.1%
*Penicillium*	*P. citrinum*	++	20–30%
	*P. chrysogenum*	+	0.1–1.0%
	*P. expansum*	+	1–5%
	*P. olsonii*	−	30–40%
	*P. polonicum*	−	0.1–1.0%
*Scedosporium*	*S. aurantiacum*	_	<0.1%
*Stemphylium*	*S. versicarium*	_	<0.1%
*Trichoderma*	*T. harzianum*	+	1–5%

^a^All samples were obtained from licensed cannabis facilities (two organically certified indoor facilities and one outdoor production facility) in the Fraser Valley of British Columbia. ^b^Mycotoxin potential: (−) Unknown; (+) Low; (++) Medium; (+++); ^C^High Relative abundance was estimated based on the relative frequency of occurrence of colonies of the particular species among the total Petri dishes assayed. Adapted from Punja and Scott (2023).

### 2.1. Phytopathogens of *Cannabis sativa*

Just like most agricultural crops, cannabis and hemp plants are grown in cultivation systems that achieve efficiency of production at reduced cost; monoculture conditions are typical for greenhouse or indoor environments or field conditions, depending on the crop. The potential sources and levels of fungal contaminants may vary depending on these environmental factors, but data are lacking on the specific similarities or differences. Hence, the quality of the cannabis and hemp products could be influenced by the specific growing environment. A large number of fungal phytopathogens are known to infect the roots, stems, leaves and inflorescences of cannabis, and hemp plants both indoors and outdoors [Punja, 2021a; Gauthier and Thiessen, 2022; United States National Fungus Collections and Fungal Database (NFCFD), 2023]. In addition, fungi considered to be epiphytes and endophytes which cause no obvious symptoms in plants have been characterized and are common in stems and roots (Figures 1, 2). Furthermore, plant organs (roots, stems, leaves, and flowers) vary in the community structure of their mycobiomes and plant pathogens (Barnett et al., 2020; Comeau et al., 2020). For example, plants grown in soil and soilless media used for indoor production may harbor different endophytic and phytopathogenic fungi (Punja and Scott, 2023). The origins of these fungi can be traced back to the growing medium used, source of plant propagation materials, or are present externally in the ambient environment. These latter populations would constitute one potential source of contaminants on cannabis inflorescences (Punja et al., 2023). Therefore, an understanding of the myriad of potential sources of contaminants on cannabis and hemp inflorescences can have an important bearing on the quality of products derived from them. This information becomes important for consumers and identifying any potential health risks.

### 2.2. Endophytes of *Cannabis sativa*

Endophytes in cannabis and hemp can be latent phytopathogens, mutualists, or commensals (Alam et al., 2021). A number of endophytes that are latent pathogens can be present as pre-and post-harvest contaminants of cannabis inflorescences (Table 1). In mutualistic responses in other plants, fungal endophytes may improve plant health by modulating responses to biotic stresses (phytopathogens and pests; Ball et al., 2006; Ownley et al., 2010; Ball et al., 2011) and abiotic factors (Kristy et al., 2022). Endophytes can produce antimicrobial compounds that may render them beneficial in the plant (Taghinasab and Jabaji, 2020; Mishra et al., 2022). There is, however, currently little published data to support these roles in cannabis or hemp plants. Paradoxically, some endophytes may also produce secondary metabolites that are harmful to humans and other animals (i.e., mycotoxins; see Section 3.3). Translocation of these mycotoxins within the plant to distal tissues, including inflorescences, has been demonstrated for other crops (Snigdha et al., 2015; Pecoraro et al., 2018; Jaster-Keller et al., 2023), but to the best of our knowledge, translocation of mycotoxins within cannabis and hemp has not been demonstrated. A secondary metabolite produced by the endophyte, *Sarocladium zeae,* inhibited biosynthesis of the mycotoxin, fumonsin, by *Fusarium verticillioides*, a phytopathogen that coexists with *S. zea* in corn seed (Gao et al., 2020). Therefore, the potential negative impacts of endophytic fungi on human health remain unknown. During post-harvest operations, however, the release of endophytic spores that can colonize the tissues of cannabis inflorescences can lead to potential increases in fungal populations that negatively influence product quality and potentially could impact consumer health (Punja et al., 2019).

### 2.3. Epiphytes of *Cannabis sativa*

Cannabis and hemp inflorescences provide unique ecological niches for fungal growth. Temperature and relative humidity conditions are higher within these tissues compared to the ambient environment, which provides a more conducive environment for fungal growth (Punja et al., 2023); however, growth may be restricted by the presence of certain terpenes in these tissues (Ranjith et al., 2021). Epiphytes on cannabis and hemp inflorescences may originate from populations of endophytes that are released from internal tissues, such as stems, during post-harvest processing of inflorescences (Punja et al., 2019). Damaged tissues can also be colonized by fungal spores that are released from harvested tissues or are present in the ambient environment during crop production. Release of nutrient compounds that favor fungal growth has not been established and would depend on the genotype of the plant. These epiphytes can grow and produce spores on damaged inflorescence tissues under favorable ambient conditions that include high temperatures and relative humidity. If sufficient growth is achieved, the potential for mycotoxin production is increased. Spores of epiphytic fungi such as *Penicillium* and *Aspergillus* species can also be observed to stick to the surface of the glandular trichome heads (Figure 2), which are produced in abundance on the inflorescence bract tissues of cannabis where terpene concentration are also high. In organic cannabis production facilities, populations of air-borne fungal propagules originating from the soil may be high, and a greater prevalence of species of fungal epiphytes has been reported on cannabis inflorescences than in conventional culture (Punja and Scott, 2023). In addition, fungal spores may be observed stuck to the surface of trichome glands or are embedded in the sticky resin, potentially enhancing these populations (Punja and Scott, 2023). The predominant terpenes found in cannabis inflorescences, namely myrcene, β-caryophyllene, limonene, α-terpinene, and α-pinene (Chacon et al., 2022) may play a role in the community structure of floral mycobiomes. Many of these compounds are shown to be fungistatic and inhibit growth of toxigenic fungi under laboratory conditions (Karpiński, 2020; Cai et al., 2022; Jiang et al., 2023). Antifungal activity of terpene essential oils may also be due to synergistic interactions of compounds. Nanoemulsions composed of terpenes of hops (*Humulus lupulus*) in which terpene profiles are similar to those in cannabis and hemp, inhibited mycelial growth, spore germination, and mycotoxin production (deoxynivalenol (DON) and its derivatives) by *F. graminearum* (Jiang et al., 2023). Complex resin composed of terpenes reduced germination of two nonpathogenic species but not that of a phytopathogen (Slinski et al., 2015). These data suggest that terpenes in various cannabis and hemp genotypes and chemotypes may limit development of some epiphytic *Aspergillus* and *Penicillium* species. More research is needed to show whether fungistasis can be overcome under conducive environmental conditions. Also, there is presently little information as to whether cannabis or hemp plants can regulate the extent of endophytic or epiphytic colonization of the inflorescence through recruitment or exclusion of microbes. This has potential significance to human health as selection for lower fungal population levels can lead to a higher quality product.

## 3. Potential health hazards of fungal contaminants in cannabis

### 3.1. Health impacts for patients with susceptible conditions

**Figure 3:**
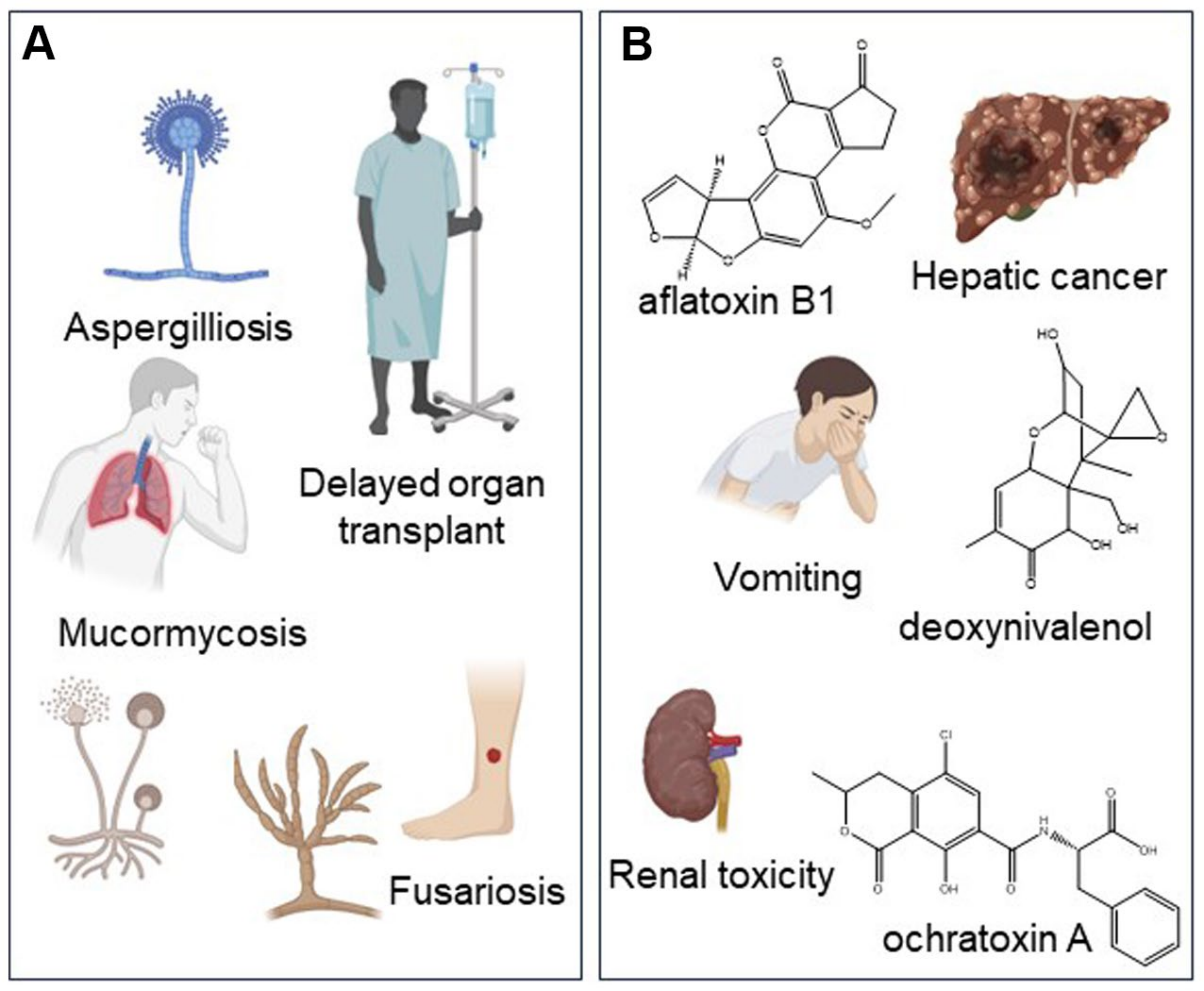
Health risks of fungal and mycotoxin contaminants to cannabis and hemp users. **(A)** Adverse health effects of fungal pathogens. *Aspergillus* was the most common organism associated with infection of cannabis users. **(B)** Adverse health risks of mycotoxins. Created with BioRender.com.

Contaminant fungi pose a recognized health risk to patients with susceptible conditions, such as those who are immunocompromised [National Academies of Sciences, Engineering, and Medicine (NASEM), 2017; Figure 3]. These fungi can cause opportunistic infections on skin and lung tissues, which could lead to life-threatening conditions. The exposure route, dosage, and frequency of cannabis and hemp use can determine the potential health effects of fungal contaminants on consumers. In patients, fungal infections were mostly associated with the smoking of cannabis (89%) and were least common for edibles (4%; Levi et al., 2019). Although the heating conditions during smoking and vaping should kill fungal spores, they may be insufficient to render all spores nonviable (Kurup et al., 1983; Szyper-Kravitz et al., 2001). At the same time, these pathogens can gain access to the airway passages as aerosols. For example, species of Mucorales (e.g., *Mucor*) can be found in cannabis inflorescences (Stone et al., 2019; Punja and Scott, 2023). These fungi release spores into the environment where they remain airborne until they enter the body through inhalation. These fungi can result in opportunistic infections on skin and lung tissues, which can lead to life-threatening conditions (see Section 3.2).

Cancer is another susceptible medical condition that can increase a patients’ risk of opportunistic infection. In 29 out of 30 US states and Washington D.C. that have legalized cannabis for recreational use, cancer is listed as a qualifying condition for medical cannabis use (Jameson et al., 2022). Cancer patients are often treated with chemotherapeutic drugs that result in compromised immunological functions. Consequently, oncologists have expressed concerns over the use of cannabis and cannabis-based products in cancer care, which is administered to improve the appetite of patients and manage symptoms of nausea, vomiting, and pain (Abrams, 2022). Several reports have shown that pulmonary aspergillosis developed in patients with malignancies or other immunocompromised states following smoking of cannabis [National Academies of Sciences, Engineering, and Medicine (NASEM), 2017]. If medical use of cannabis becomes more widely accepted, contaminant fungi represent a potential concern in public health.

Transplant surgeons have expressed concern about the use of cannabis by their patients (Levi et al., 2019; Ryan et al., 2019; Olt et al., 2021). In a study involving 52,689 Medicare kidney transplant recipients, those with post-transplant cannabis dependence or abuse (CDOA) were associated with a higher rate of all-cause graft failure (26.8 vs. 12.6%) and death (10.6 vs. 7.9%) occurring 1–3 years post-transplant as opposed to those without CDOA (Alhamad et al., 2019). Cannabis usage was associated with fungal infections in kidney transplant patients and an increased rate of aspiration pneumonia (4.3 vs. 1.1%) and other forms of pneumonia (18.3 vs. 10.6%; Alhamad et al., 2019; Olt et al., 2021). *Aspergillus* was the most common organism followed by Mucorales (includes *Mucor* spp.) responsible for these infections (Levi et al., 2019; Olt et al., 2021). The Canadian Cardiovascular Society/Canadian Cardiac Transplant Network has made a strong recommendation for a 6-month abstinence from cannabis use before heart transplant (Chih et al., 2020). Cannabidiol has been studied as a potential preventative agent to reduce COVID-19 viral load and to suppress cytokine storms related to viremia (Nguyen et al., 2022). COVID 19 patients are also immunocompromised and 20–30% of them get aspergillosis (Kuehn, 2021; Lin et al., 2022; Feys et al., 2023). Given the prevalence of COVID 19 infection, fungal contaminants in cannabis and hemp can be important public health concern in preventative use.

### 3.2. Fungal pathogens directly affecting humans

A number of studies have examined the allergenic responses to *Aspergillus* spp. and aspergillosis associated with cannabis use. Trullas et al. (2010) reviewed seven case reports of aspergillosis in cannabis smokers. Cases not reported in Trullas et al. (2010) are summarized in Table 2. Pulmonary aspergillosis is a common form of *Aspergillus* infection and has been reported in cannabis users with HIV and type 1 diabetes (Remington et al., 2015; Salam and Pozniak, 2017). *Aspergillus fumigatus* is a common species associated with aspergillosis in cannabis users (Table 2). It was also cultured from the cannabis products used by patients with aspergillosis (Kagen et al., 1983; Kouevidjin et al., 2003).

**Table 2:** Reports of allergenic responses to toxigenic fungi and mycoses associated with cannabis users (excluding those reported in Trullas et al., 2010).

	Species	Diagnosis reported/Study description; Use; Immunosuppressive	Fungal identification method	References
*Aspergillus*	*A. fumigatus*	Allergenic response in 28 cannabis smokers; one with systemic aspergillosis, seven with bronchospasm after smoking	Patient tissue samples—Serology	Kagen et al. (1983)
	*A. flavus*		Cultured from products (serology)	
	*A. niger*			
	*A. fumigatus*	Bronchopulmonary aspergillosis; cannabis smoker; and asthma	Patient tissue samples—Serology	Kouevidjin et al. (2003)
			Cultured from products	
	*A. flavus*			
	*A. fumigatus*	Invasive pulmonary aspergillosis; metastatic colorectal cancer and chemotherapy	Sequencing	Cescon et al. (2008)
	*A. fumigatus*	Chronic pulmonary aspergillosis; daily cannabis smoker; with steroid use	Serology	Gargani et al. (2011)
		Chronic pulmonary aspergillosis; daily cannabis smoker	Serology	Gargani et al. (2011)
	*A. rugolosa*	Chronic necrotizing pulmonary aspergillosis; cannabis via vaporizer daily; diabetes	*A. rugolosa* confirmed by sequencing	Remington et al. (2015)
	*A. fumigatus*		*A. fumigatus* (cultured from patient)	
	*Penicillium* sp.			
	*A. fumigatus*	Disseminated aspergillosis; former cannabis user; HIV + steroids	Cultured from patient	Salam and Pozniak (2017)
*Fusarium*	*Fusarium* sp.	Cutaneous fusariosis; hyper-IgE-syndrome + steroids; cannabis grower^*^	Cultured from patient (microscopy)	Altibi et al. (2020)
*Alternaria*	*Al. alternata*	Allergenic responses in 316 (11.8% of participants) current cannabis users	Serology	Min and Min (2018)
*Cryptococcus*	*Cryptococcus* sp., including *C. neoformans*	Cryptococcal meningitis; daily cannabis smoker; and no evidence of immunodeficiency	Patient cerebrospinal fluid samples—Microscopy and mass spectrometry	Shapiro et al. (2018)

^*^Patient’s cannabis plants “died out because of the mold.”

Cannabis has also been reported to contain fungi in the order Mucorales (including *Mucor* spp.; Stone et al., 2019; Punja and Scott, 2023). These fungi release spores into the environment where they remain airborne and can potentially enter the body through inhalation. Pulmonary mycormycosis was reported seen in patients in a diabetic patient (Stone et al., 2019).

At present, only one published study has examined the association between cannabis use and fungal infection using data collected nationally (Benedict et al., 2020). From the United States insurance records from 53,217 people in 2016, the study demonstrated that cannabis users, particularly those with susceptible conditions, were 3.5 times more likely to develop a fungal infection than non-users. Aspergillosis accounted for 43% of fungal infections among cannabis users, while mucormycosis accounted for 3%. Among the population that was examined, individuals who used cannabis and had fungal infections were generally immunocompromised, a greater proportion were males, and a higher percentage also used tobacco when compared to those that did not use cannabis (Benedict et al., 2020). The source of the infections could not be determined to have originated specifically from cannabis products.

## 4. Mycotoxins in cannabis and hemp

Among several fungal species reported to be present on cannabis and hemp inflorescences (Table 1), a number can produce mycotoxins when grown on culture medium and potentially in the affected plants (Tables 3–5). Since fungal growth precedes mycotoxin production, generally the greater the growth, the higher the predicted levels of mycotoxins (Ismaiel and Papenbrock, 2015). Fungal growth on any substrate is directly impacted by water activity of the substrate (a_w_) and most fungi grow best at a_w_ of 0.83–0.99 (Caplan et al., 2022). An important caveat, however, is that presence and recovery of toxigenic fungi from cannabis inflorescences in routine laboratory testing mandated by governmental agencies should not imply that they produce mycotoxins unless the moisture and temperature requirements for extended growth and mycotoxin production are available, which may differ by the type of mycotoxins produced (Milani, 2013). On cannabis inflorescences, visible fungal growth may occur prior to or after harvest (Figure 1). Post-harvest growth commonly leads to rejection of final products due to visible mold contamination and these buds are destroyed (Punja, 2021d). Subsequently, cannabis inflorescences that are improperly dried or stored under humid conditions may be expected to raise concerns for mycotoxin accumulation. If sufficient drying is achieved, the a_w_ (water activity) of cannabis tissues is reduced to <0.7, which prevents the growth of most fungi (Carter, 2019). Because two of the most commonly encountered fungi that can potentially produce mycotoxins in cannabis tissues, namely *Penicillium* and *Aspergillus* spp., can survive at a_w_ of 0.62–0.7, this range is suboptimal for fungal growth. Additionally, environmental conditions for gene transcriptional activity for mycotoxin production are narrower than those for fungal growth (Kolawole et al., 2021). Therefore, it is improbable that mycotoxin levels would significantly increase in cannabis tissues maintained at a low a_w_. The drying phase can be a component of quality assurance that can reduce the concerns for accumulation of mycotoxin produced by epiphytes in cannabis products.

**Table 3:** Species of *Aspergillus* that have been reported in *Cannabis sativa* and mycotoxins produced *in vitro*.^a^

*Aspergillus* subgenus^b^	*Species*	Aflatoxins (B_1_, B_2_, G_1_, G_2_, and M_1_) and sterigmatocystin	Ochratoxins	Patulin	Gliotoxin	Trichothecenes and Fumonisin (B_2_, B_4_, and B_6_)	Other ^c^	Source
Nidulantes	*A. nidulans*	+					+	Zingales et al. (2020); Zhao et al. (2021)
	*A. unguis*	+					+	Reijula and Tuomi (2003)
	*A. versicolor*	+	+	+		+	+	Frisvad (2018); Zingales et al. (2020)
	*A. sydowii*		+	+			+	Frisvad (2018); Steenwyk et al. (2019)
Circumdati	*A. flavus*	+		+	+		+	Frisvad et al. (2019); Navale et al. (2021)
	*A. parasiticus*	+		+		+		Frisvad et al. (2019); Gil-Serna et al. (2020)
	*A. ochraceous*		+				+	Varga et al. (2009); Hareeri et al. (2022)
	*A. sclerotiorum*		+					Palumbo et al. (2007)
	*A. terreus*			+^d^	+	+	+	Yin et al. (2016); Frisvad (2018) Lass-Flörl et al. (2021)
	*A. niger*		+		+	+	+	Frisvad et al. (2018); Chen et al. (2023)
	*A. candidus*	+		+			+	Fraga et al. (2008); Frisvad (2018)
Fumigati	*A. fumigatus*		+	+	+		+	Shankar et al. (2018); Steenwyk et al. (2020); Chen et al. (2023)
Cremei	*A. wentii*		+					Díaz et al. (2009)
Aspergillus	*A. restrictus*						+	Brandhorst et al. (1996)
	*A. penicilliodes*						+	Micheluz et al. (2016)

^a^Pathogens reported for *Cannabis sativa* (Sources: United States Fungus-Host Database, Gautam et al., 2013; Kusari et al., 2013; Punja et al., 2019; Punja, 2021a,b,c,d).

^b^Assignment to subgenus based on Houbraken et al. (2020).

^c^Other includes selected secondary metabolites include but not limited to: aspyridone (*A. nidulans*); versicolorin (*A. versicolor*); aflatrems and cyclopiazonic acids (*A. flavus*); citrinin (*A. fumigatus* and *A. terreus*); secalonic acid (*A. flavus* and *A. fumigatus*), and oxalic acid (*A. niger*), terrain, lovastatin, and terretonin (*A. terreus*); fumagillin and fumitremorgins (*A. fumigatus*); asphenamate and indole alkaloids (*A. restrictus*); asperglaucide (*A. penicilliodes*).

^d^Production of patulin by *A. terreus* has been reported but is called into question by Frisvad, 2018.

**Table 4:** Potential for toxigenic *Penicillium* spp. isolated from *Cannabis sativa* to synthesize toxic secondary metabolites.

*Penicillium* subgenus	Species^a^	Ochratoxins	Citrinin	Patulin	Mycophenolic Acid	Alkaloids	Other^b^	Source
Aspergilliodes	*P. glabrum*			**+**	**+**			Mahmoudian et al. (2021); Bokhari and Aly (2009)
	*P. citrinum*	**+**	**+**	**+**			**+**	Barkai-Golan (2008); Gherbawy and Shebany (2018); Kamle et al. (2022)
	*P. copticola*						**+**	Ezekiel et al. (2020)
	*P. paxilli*					**+**		Pitt (2002); Bräse et al. (2009)
	*P. sumatrense*						+	Malmstrøm et al. (2000)
	*P. daleae*						**+**	Frisvad and Filtenborg (1990)
	*P. oxalicum*					**+**		Kim et al. (2012); da Silva-Filho et al. (2021)
	*P. simplicissimum*					**+**	**+**	Bräse et al. (2009); Dai et al. (2020)
	*P. corylophilum*		**+**					dos Santos et al. (2012)
Penicillium	*P. expansum*		**+**	**+**		**+**	**+**	Assaf et al. (2020); Li et al. (2020)
	*P. griseofulvum*			**+**			**+**	Banani et al. (2016); Bräse et al. (2009)
	*P. olsonii*	**+** ^cd^		**+** ^d^		**+** ^d^	**+**	Wang et al. (2016); Soliman et al. (2021)
	*P. brevicompactum*	**+** ^c^		**+**	**+**		**+**	Pitt (2002); Wang et al. (2016)
	*P. spathulatum*	**+**					**+**	Frisvad et al. (2013); Wang et al. (2016)
	*P. chrysogenum*	**+**				**+**	**+**	Alapont et al. (2014); Bräse et al. (2009)
	*P. polonicum*	**+**				**+**	**+**	Alapont et al. (2014); Bräse et al. (2009)
*Circumdati*	*P. sclerotiorum*		**+** ^c^	**+** ^c^				Yin et al. (2017)

^a^Pathogens listed in United States fungus-host database.

^b^Other includes but it is not limited to toxins and other important secondary metabolites: Alternaria toxin (*P. chrysogenum, P. expansum, P. griseofulvum*), asperphenamate (*P. brevicompatum, P. olsonii, P. spathulatum*), asperentin, citreovidin (*P. citrinum*), curvularin (*P. sumatrense*); glandicolin and meleagrin (*P. oxalicum*); griseofulvin, yanuthone D (*P. griseofulvum*), cyclopiazonic acid (*P. griseofulvum, P. polonicum*), penicillin (*P. expansum, P.chrysogenum*), moniliformin and sporogen AO1 (*P. copticola*), and, PR-Toxin (*P. chyrsogenum*); roquefortines (*P. griseofulvum, P. oxalicum*); tremogens (*P. olsonii, P. simplissimum and P. expansum*).

^c^Produced only at low levels

^d^Biosynthesis genes were detected but not toxin.

**Table 5:** Potential for mycotoxin production by species of *Fusarium* reported in *Cannabis sativa*.

*Fusarium* species^abc^	Trichothecenes					Emerging ionophores			Other emerging mycotoxins						Fumonisins
	3A DON ^d^	15A DON	4,15-diNIV	DAS	T-2	BEA	BIK	ENB	BUT	CUL	FA	MBO	MON	ZEA	FB_1_ + FB_2_ + FB_3_
*F. avenaceum*								**+**					**+**		
*F. brachygibbosum*				**+**	**+**	**+**									
*F. equisiti*			**+**	**+**											
*F. falciforme*															
*F. graminearum*	**+**	**+**				**+**			**+**	**+**				**+**	
*F. lichenicola*															
*F. oxysporum*						**+**	**+**	**+**					**+**		
*F. proliferatum*						**+**	**+**	**+**				**+**	**+**		**+**
*F. solani*						**+**									
*F. sporotrichioides*					**+**	**+**			**+**						

^a^Pathogens described in Gwinn et al. (2022). ^b^*F. avenaceum* was recently described in hemp (Smith et al., 2023). ^c^Mycotoxin production is based on analysis of strains of the fungi that were not isolated from cannabis (O'Donnell et al., 2018). ^d^3ADON, 3-Acetyldeoxynivalenol; 15ADON, 15-Acetyldeoxynivalenol; 4, 15-diNIV, 4, 15-Diacetylnivalenol; 8-ANEO, 8-Acetylneosolaniol; BEA, Beauvericin; BIK, Bikaverin; BUT, Butenolide; CUL, Culmorin; DAS, Diacetoyxyscirpinol; ENB, Enniatin B; FA, Fusaric acid; FB, B fumonisins; MBO, 8-O-methylbostrycoidin; MON, Moniliformin; NIV, Nivalenol; T-2, T-2 toxin; and ZEA, Zearalenone.

### 4.1. Mycotoxin-producing fungi

**Figure 4:**
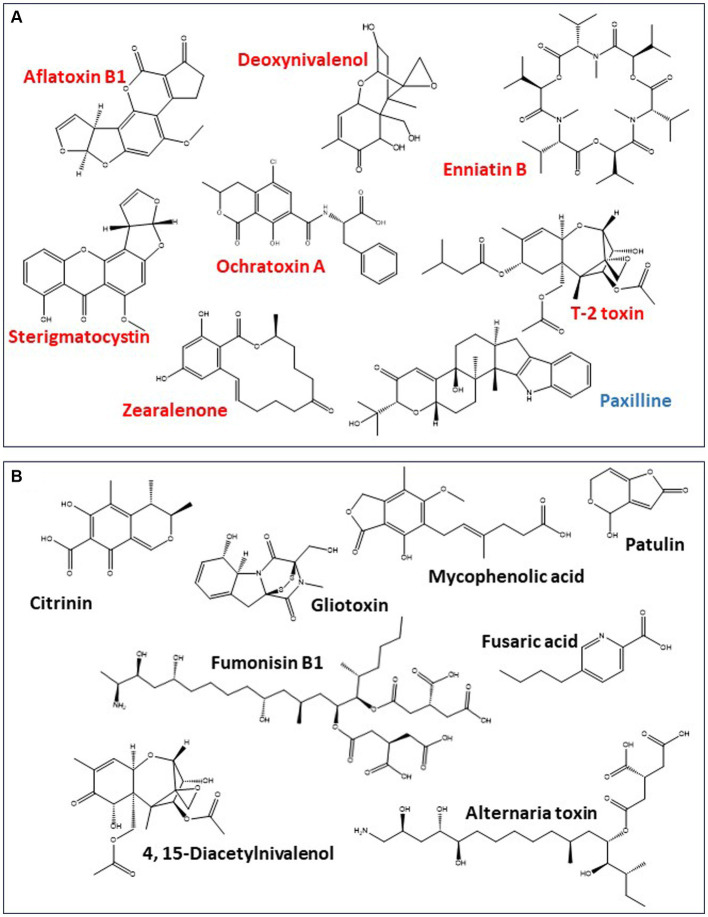
Mycotoxin production potential of toxigenic fungi reported in cannabis mycobiome. **(A)** Mycotoxins that have been reported in cannabis and hemp products (red) or for which genes for their production have been reported in toxigenic fungi isolated from cannabis products (blue). **(B)** Other mycotoxins that are known to be produced by toxigenic fungi associated with cannabis and hemp but have not been reported in the crops or their commercial products.

Fungi reported to be present in cannabis and hemp plants (i.e., *Penicillium, Aspergillus,* and *Fusarium* spp.) have the genetic potential to produce a range of mycotoxins, including ones recognized as significant problems on other agricultural crops [e.g., aflatoxins, ochratoxin A (OTA), DON, fumonisins (FUM), zearalenone (ZEA), T-2 toxin, HT-2 toxin, citrinin (CIT), and patulin (PAT); Figure 4]. Some mycotoxins such as aflatoxins are produced by a few species, whereas others (e.g., OTA and PAT) are produced by multiple species belonging to multiple genera. The synthesis of mycotoxins involves several metabolic pathways and corresponding enzymes, such as polyketide synthases, nonribosomal peptide synthetase, the mevalonate pathway, or a combination of these. Mycotoxin profile, however, is typically different among genera. For example, *Penicillium* and *Aspergillus* are members of the same family, yet they only have a few secondary metabolites in common (Samson et al., 2011), and their mycotoxin profiles are very different from other members of the cannabis floral mycobiome. Other fungi such as *Alternaria, Cladosporium, Chaetomium, Mucor,* and *Trichoderma* that can be found in cannabis inflorescences (Table 1) may also produce different cytotoxic and genotoxic mycotoxins such as PAT, alternariol, tenuazonic acid (Ismail et al., 2023), chaetoglobosins (Fogle et al., 2008), viridin, and gliotoxin (Sugui et al., 2007; Bulgari et al., 2020) when adequate fungal growth has been achieved. This list is not exhaustive but is reflective of the diversity of the floral mycobiome from a few recent studies (Punja et al., 2023). Under organic crop production systems, the diversity of fungal species present on cannabis inflorescences can be greater than in conventional production systems (Punja and Scott, 2023). It is important to note that fungi listed in Table 1 are commonly found on a wide range of plant species and, hence, are not unique to cannabis and hemp. They pose a potential concern to cannabis consumers only if they have the potential for mycotoxin production or have been shown to infect individuals as discussed in Section 3.

### 4.2. *Aspergillus* and *Penicillium* mycotoxins

There is a paucity of published studies on mycotoxin presence in cannabis-derived products (Table 6). Most of these studies included a limited number of samples (*n* < 100), with most of them identified as illicit samples. The legalization of cannabis in several countries, including Canada and in numerous US states, should increase the availability of samples for testing for mycotoxin presence moving forward. Species of *Aspergillus* are known to produce aflatoxins and OTA (Table 3). Aflatoxins B1, B2, G1, and G2 represent some of the most toxic and carcinogenic compounds occurring in nature. Although aflatoxins were produced by *A. flavus* and *A. parasiticus* when cultured on sterile herbal cannabis samples (Llewellyn and O'Rear, 1977), they have rarely been identified in compliance testing (Jameson et al., 2022; Table 6). This anomaly may be due to: (1) predominance of atoxigenic isolates in cannabis and hemp production systems (Sweany et al., 2022); (2) environmental conditions during crop production that do not support growth and development of the fungus to the extent where mycotoxins accumulate; (3) terpenes found in the inflorescence that can restrict fungal growth and development (see section 2.2); or (4) degradation of compounds by the plant or its associated microbes can occur. While the presence of potentially toxigenic species of *Aspergillus* has been reported on cannabis inflorescences (Table 1), there are fewer reports confirming the production of their associated mycotoxins (Table 6). Further research is needed to demonstrate whether the current concern over *Aspergillus* contamination of cannabis flowers can or should be extrapolated to infer the presence of associated mycotoxins.

**Table 6:** Reports of toxigenic fungi in cannabis and cannabis products sold for consumer consumption or consumed by patients with mycoses.

Toxigenic fungus or mycotoxin detected^a^	Type of sample	Region or Country State, City	Detection method^b^	Results	References
*A. fumigatus; A. flavus; A. niger;* and *Penicillium* spp.	Herbal cannabis smoked by subjects (*n* = 14)	USA—WI—Milwaukee	C-NA	93% of samples contained at least one species; lit and unlit cigarettes allowed passage of spores	Kagen et al. (1983)
*A. glaucus*; *A. restrictus*; and *A. flavus*	Seized samples (*n* = 25 for fungi; *n* = 52 for mycotoxins)	USA—DC MD, VA	C-MIC 2D-TLC	Out of fifteen species, the three most common *Aspergillus* species were: *A. glaucus* (96% of samples); *A. restrictus* (80%), and *A. flavus* (72%). *Penicillium citrinum* was isolated from 40% of samples. Aflatoxins—inconclusive; reported as Aflatoxin B1-like compounds	Krawczeniuk et al. (1987)
*A. fumigatus A. flavus*	Herbal cannabis smoked by symptomatic patient		C-NA		Kouevidjin et al. (2003)
Primary toxigenic fungi in at least one sample: *A. terreus; A. versicolor*; *P. citrinum;* and *P. paxilli*	Purchased in dispensaries (*n* = 10)	USA—CA (six samples); Amsterdam (four samples)	MICB	PAX biosynthesis genes identified in at least one sample; method does not discriminate between viable and nonviable fungi	McKernan et al. (2015)
*Aspergillus* spp.	Street samples—illicit resin (*n* = 90)	Europe (Madrid)	C-MIC	10% of samples contaminated with *Aspergillus* sp.	Pérez-Moreno et al. (2019)
Aflatoxin B1	Purchased from small retailers (*n* = 14)	Europe	ELISA	50% of products higher in aflatoxin B1 than EU limits	di Nardo et al. (2020)
T-2, ZAN, ZEN, ENNB1, ENNA, and ENNA1	CBD gelatin capsules (*n* = 10)	Europe	UHPLC-Q-Orbitrap HRMS	Co-occurrence of two or more mycotoxins in 40% of samples	Narváez et al. (2020)
STM	Seized street samples (*n* = 93)	Austria	LC–MS/MS	STM—29% samples; no aflatoxin B1	Stempfer et al. (2021)
OTA	Seized street samples (*n* = 142)	Luxembourg	HPLC-FLD	No aflatoxins; OTA—39% of resin samples and 27% of herbal samples	Buchicchio et al. (2022)
Aflatoxins, OTA	Compliance tested samples (*n* = 5,654 flowers) and (*n* = 3,760 for extract)	USA—CA	LC-MS/MS	Aflatoxins higher than CA limits in one sample; no detection of OTA	Jameson et al. (2022)

^a^Mycotoxin abbreviations: STM, Sterigmatocystin (precursor in aflatoxin pathway); OTA, Ochratoxin A; PAX, Paxilline. ^b^Method abbreviations: C-NA, Culture with identification method not described; C-MIC, Culture with identification by microscopsy; MICB-MLC-MS/MS, Non-targeted liquid chromatography.

The most frequently recovered toxigenic fungi from cannabis tissues are *Penicillium* spp. (Punja et al., 2019, 2023; Punja, 2021d). They can produce citrinin, OTA, CIT, and PAT, but only OTA has been found in cannabis samples to date (Tables 4, 6). Exposure of humans to OTA can result in kidney damage (Bui-Klimke and Wu, 2015). One fungal species associated with cannabis, *Talaromyces pinophilus*, produced OTA in culture, while a closely related strain, *T. radicus*, did not (Laut et al., 2023). Although no studies have compared mycotoxin levels in legalized and illicit cannabis products, the literature suggests that OTA presence can be of concern in illicit cannabis. In a recent study, OTA was detected in one-third of 142 illicit cannabis samples (Buchicchio et al., 2022). In a second study, OTA was not detected in more than 9,000 flower and extract samples from ~300 legal cannabis producers and manufacturers for compliance testing in California during 2020 and 2021 (Jameson et al., 2022). This study suggests that legalization of cannabis production may be having an intended consequence of improving quality with lower mold contamination, but additional studies are needed to confirm this.

In cannabis and hemp products that are smoked or vaped, aflatoxin B1 is likely to be degraded since it was completely degraded under experimental conditions at 180°C (Raters and Matissek, 2008). Temperatures in marijuana cigarettes can exceed 600°C (Fehr and Kalant, 1972) while coil temperature of three types of vaporizers exceeded 400°C (Oar et al., 2022). For example, OTA levels in dry wheat were reduced by 25% when exposed to 200°C for 6 min and by 44% if exposed for 12 min (Boudra et al., 1995). The degradation of these mycotoxins by heat does not always result in reduced toxicity since some degradation products are as toxic as the parent molecule (Kabak, 2009). Further studies are needed to confirm relationships between modes of product preparation and consumption and mycotoxin, and, in particular, potential for accumulation of mycotoxins in concentrated cannabis and hemp extracts used to prepare consumable items such as infused gummies and candy, or in capsules and gels, warrants further study.

### 4.3. *Fusarium* mycotoxins

Production of mycotoxins has been associated with several *Fusarium* species (Table 5). A number of *Fusarium* spp. that have been reported to infect cannabis inflorescences can produce an abundance of mycelial growth and mycotoxins under laboratory conditions (Gwinn et al., 2022; Munir et al., 2023). In a recent survey, diseases caused by *Fusarium* (including *Fusarium* head blight and *Fusarium* wilt) were commonly encountered during cannabis and hemp production in the United States (Munir et al., 2023). This may have important implications for cannabis worker safety, as exposure to infected cannabis plants has been linked to cutaneous fusariosis (Altibi et al., 2020). At present, there is a scarcity of analytical studies on *Fusarium* mycotoxin accumulation in cannabis and hemp inflorescences. There is only one reported study of DON production in hemp (Bergstrom et al., 2019, 2020). In that study, DON was present in the grain and floral tissues of outdoor-grown hemp at concentrations of up to 7 ppm, which was greater than the 1 ppm level established by the U.S. Food and Drug Administration permitted for cereal grains used for food [United States Food and Drug Administration (FDA), 2010]. *Fusarium* mycotoxins, including trichothecenes, ZEA, ionophores, FUM, and fusaric acid, have both acute and chronic effects in humans (Gruber-Dorninger et al., 2017; Ji et al., 2019). In addition to causing chronic immunosuppression and cancer, trichothecenes such as DON (one of the most common *Fusarium* mycotoxins, also known as vomitoxin), nivalenol (NIV), and T-2 toxin can cause acute symptoms of toxicosis, including nausea, vomiting, diarrhea, and death (Ji et al., 2019). Translocation of DON from stem base infections to the inflorescences has been reported in barley and wheat plants, which while not showing obvious symptoms in the inflorescences, may contain DON or other mycotoxins (Pecoraro et al., 2018). An analysis of gelatin capsules containing the cannabinoid, cannabidiol (CBD; *n* = 10) revealed the presence of T-2 toxin in 10% of the samples but the levels were below those allowed for animal food (Narváez et al., 2020). *Fusarium* mycotoxins are generally considered to be thermally stable and can still be detectable in foods that have undergone thermal processing (Bretz et al., 2006; Kabak, 2009). We are unaware of studies that have discussed the fate of these mycotoxins in combustion. When cannabis or hemp is smoked in a water pipe, DON contamination may be reduced because DON leaches into water at high temperatures; the water acts as filteration for the smoke (Liu et al., 2019; Feizollahi and Roopesh, 2022). However, even though the smoking process can reduce the level of *Fusarium* mycotoxins, these mycotoxins are absorbed into the bloodstream efficiently and rapidly through inhalation. Further studies are needed to demonstrate if *Fusarium* mycotoxins could pose potential health risks to consumers, which will be dependent on variables such as the growing environment to which the cannabis and hemp plants are exposed, as well as post-harvest processing and manufacturing and the form of exposure to consumers.

Cannabis hyperemesis syndrome (CHS) is a condition of recurring vomiting that some individuals develop after prolonged cannabis use (Perisetti et al., 2020). In Colorado, most cannabis-related emergency department (ED) visits were for CHS (Wang et al., 2021). The etiology of CHS is largely unknown and it may be triggered by multiple chemicals present in cannabis. Although no studies have linked presence of *Fusarium* mycotoxins (or their degradation products) in cannabis to CHS, the symptoms of CHS are remarkably similar to the toxicity of DON, NIV, and T-2 toxin, all of which can result in vomiting and partial or complete refusal of food (Bonnet et al., 2012). Additionally, the ingestion of fusaric acid—another common *Fusarium* mycotoxin that is often found together with DON—can cause hypotension (Gruber-Dorninger et al., 2017). This is consistent with the observation that CHS can be relieved by hot water bathing. Further studies are needed to establish the scale of contamination cannabis and cannabis products by *Fusarium* spp. and to understand neurotoxicity of potential mixtures of cannabinoids with *Fusarium* mycotoxins. This is especially important given the presence of several *Fusarium* species in cannabis and hemp inflorescences, both in indoor and outdoor cultivation (Punja, 2021a, 2021b; Punja and Scott, 2023).

Zearalenone is another prevalent *Fusarium* mycotoxin that is a major threat to food safety in cereal crops. It was isolated from 60% of CBD capsules (Narváez et al., 2020), but at levels below limits established for food products (Ji et al., 2019). Because of its structural similarity to estrogen, ZEA binds to estrogen receptors and affects reproductive systems of animals. This can potentially add to the adverse health effects associated with cannabis use in a subsegment of the population, which is an increased risk of preterm birth and small-for-gestational-age infants in prenatal exposure (Ryan et al., 2021). In rodent models and human cells, ZEA is immunotoxic, induces toxicity in the liver and kidneys, and is hemotoxic (Choi et al., 2012; Ji et al., 2019). Findings to date suggest that levels of certain mycotoxins associated with *Fusarium* species infecting growing cannabis or hemp plants pre-harvest may be found in products where extraction and concentration of the mycotoxins can occur alongside the cannabinoids of interest.

### 4.4. Other potential mycotoxins

The fungi belonging to the Mucorales produce mucoricin, a ricin-like toxin that damages host cells *in vitro* by inhibiting protein synthesis and is required for pathogenesis (Soliman et al., 2021). Enniatins and beauvericin are cyclodepsipeptides that are often classified as emerging mycotoxins because they are not regulated, and thus, there is little routine testing conducted for them. In animal studies, BEA, ENN, and MON can cause reproductive disruption in both males and females and increased offspring mortality (Chiminelli et al., 2022). In the study on CBD gelatin capsules, one sample contained three enniatins at levels similar to those of ZEA (Narváez et al., 2020). The U.S. EPA and Health Canada have registered a number of biopesticides for cannabis and hemp cultivation that contain *Trichoderma* spp. as active ingredients [United States Environmental Protection Agency (US EPA), 2023]. These species, including *T. harzianum, T. virens,* and *T. asperellum*, can all colonize cannabis plants as an endophyte (Punja and Scott, 2023; Scott and Punja, 2023), and in culture produce toxins, such as viridin and gliotoxin, that could potentially impact consumer health. Gliotoxin is a powerful mycotoxin that inhibits growth of other fungi and causes cytotoxicity by suppressing macrophage immune function and inflammation (Sugui et al., 2007). Additional research is required to establish whether these secondary metabolites are produced in cannabis inflorescence tissues following application of these beneficial fungi for plant pathogen control during the crop production cycle.

## 5. Contaminant regulation and assessment for cannabis and hemp

ASTM International, AOAC International, and U.S. Pharmacopeia are presently developing standardized methods for quantification of fungal and mycotoxin contaminants in cannabis and hemp, but there is there is a paucity of data available to determine the prevalence of these contaminants and their health impacts to cannabis and hemp consumers worldwide. The regulations currently in place have been largely adopted from the food industry, where fungal and yeast contamination of food products consumed by humans is not uncommon and can result in illnesses and potential fatalities. While the latter has yet to be studied in a statistical robust matter to support human health risk assessment of cannabis and hemp products, regulatory policies and assessment technology are evolving to meet the production and safety needs of the emerging industries.

### 5.1. Contaminant regulation for cannabis and hemp worldwide

The current regulatory policies for assessing cannabis contaminants in the United States and European Union have been extensively reviewed (Pruyn et al., 2022; Veit, 2023; Jameson et al., 2022). In the United States, the regulation of fungal contaminants in CBD and hemp products falls under the jurisdiction of federal agencies following the passage of the 2018 Farm Bill. However, due to the illegal status of cannabis at the federal level in the United States, the contaminant regulation policies for cannabis and cannabis-related products fall under the jurisdiction of state agencies. Each legalized state therefore has its own regulatory policies and testing programs (Jameson et al., 2022; Pruyn et al., 2022). This creates a dilemma for consumers due to varied potential health exposure risks. For example, regulatory action levels exist for aflatoxins and ochratoxins in some US states, but the levels are highly variable, ranging from a zero-tolerance policy in some states to no action level in others (Jameson et al., 2022). Similarly, the quantification of total yeasts and molds (TYM) and regulatory action levels are highly variable by state. Paradoxically, in jurisdictions where cannabis is still considered illegal but is being sold, there are no regulations to limit fungal contaminants in products. Not surprisingly, a few studies have found that illicit cannabis samples contained more contaminants than legally grown cannabis (Eykelbosh, 2021; Southall, 2022; Gagnon et al., 2023). The literature also suggests that aflatoxins and OTA can be more prevalent in illicit cannabis samples (see Section 4.2). These observations point to the need to harmonize the regulatory action levels for TYM and mycotoxin contaminants across all legalized jurisdictions for cannabis production. It also underlies the importance of supporting legal cannabis and hemp production systems where stringent regulations ensure a higher quality product.

Worldwide, current cannabis contaminant regulations have been directed to address a combination of concerns stemming from production, quality, and safety issues. The European Pharmacopoeia Commission (2023) provides generalized guidelines for aflatoxin and ochratoxin testing, and each member of the European Union has its own interpretation of these requirements. Countries such as Germany and the Netherlands have implemented individual monographs for cannabis (Veit, 2023). A few non-E.U. countries, such as Canada and Israel, also follow the general guidelines for aflatoxin and ochratoxin testing in the European Pharmacopoeia [Israel Ministry of Health—Medical Cannabis Unit (IMC), 2017; Health Canada, 2020]. Canada has an irradiation requirement for the export of cannabis to other countries. In Israel, cannabis is irradiated (beta and gamma) before it can be used for medical purposes. No countries, or any state in the United States, currently require testing for presence of *Fusarium* spp. or their mycotoxins.

### 5.2. Methods for assessment of molds and human pathogens

The general guidelines established by AOAC International and United States Pharmacopeia for microbial contamination include testing methods based on isolation on culture media and enumeration of colonies (i.e., total yeast and mold, or TYM) and identification by quantitative PCR (qPCR) analysis, but other methods based on molecular technology are in development (Goldman et al., 2021). Fungi that are known to be pathogens of humans require a qualitative presence/absence analysis. All methods are potentially problematic for regulatory agencies. For example, using culture-based methods to detect *Aspergillu*s spp. can be difficult due to the problems in differentiating human pathogenic and nonpathogenic species. There is currently no requirement to identify the colonies to species level. While it often requires a highly trained mycologist to correctly identify these fungi to species, it is essential in evaluating them for human health risks (Chen et al., 2019). In addition, multiple species of the same genus can infect plants of cannabis and hemp. For example, in Kentucky, *Fusarium avenaceum,* members of the *F. incarnatum-equiseti* species complex*, F. sporotrichioides,* and *F. graminearum* were all isolated from hemp samples collected during a single cropping season (Smith et al., 2023). This illustrates the importance of species identification from TYM testing.

Currently, TYM quantification is widely used by regulatory agencies such as Health Canada and different state regulatory agencies in the United States. The regulatory action levels for TYM can vary considerably by jurisdiction, ranging from 10,000 colony forming units per gram (cfu/g) to 100,000 cfu/g. Also, there is no standard dilution curve that can be used to measure test reliability because of the inability to ship cannabis samples across state lines. The assessments are usually conducted by certified commercial laboratories using plating assays that homogenize and plate extracts from tissues onto different agar media and enumerate colonies after 3–5 days of incubation. Since the community structure of the mycobiome varies according to season and is impacted by other environmental factors, such as temperature, humidity, and air movement, there is considerable biological variability among samples in TYM content (Punja et al., 2023). Furthermore, the TYM assessments are based on enumeration of all yeasts and molds present in a sample, regardless of whether they have a previous history for concern based on human exposure or have potential for mycotoxin production. For example, cannabis plants that are treated with a registered fungal biological control agents (e.g., *Trichoderma* spp.) would likely carry over sufficient propagules in the inflorescence tissues that cause it to fail to pass regulatory limits in certain jurisdictions (Punja et al., 2023). The current concept of TYM testing requires further evaluation, and determination of its value to provide information on the fungal species composition to support human health risk assessment is an area that urgently needs assessment.

The variability in the regulatory action levels based on TYM and the inter-laboratory variation of testing results bring into question how cannabis producers, consumers, and health practitioners can reliably interpret what is safe from what is not. More targeted approaches such as testing specifically for certain fungi known to pose a threat to consumers due to potential mycotoxin production (e.g., *Aspergillus* spp. and *Fusarium* spp.) would be more informative when compared to an assessment of total fungi and yeasts present. This is consistent with the practices currently used for regulation and monitoring of agricultural food products consumed by humans. If similar TYM testing were to be imposed on these foods, many could fail to pass due to their natural inherent microbiome composition. In fact, a subset of the microbiota of cannabis inflorescences may be providing undetermined benefits based on what is known from other crop plants (Punja et al., 2023).

Several toxigenic *Penicillium* and *Aspergillus* species were detected on dispensary-based cannabis flowers, including *P. citrinum* and *P. paxilli*, when samples were analyzed by next-generation sequencing but not by culture-based methods (McKernan et al., 2015). Molecular methods such as next-generation sequencing and qPCR, depending on the primer/probe, are far more sensitive and specific than culture-based methods (Sarma et al., 2020). For DNA-based testing, it is recommended that the enrichment of cannabis matrices in fungus-specific media requires at least 48 h for *Aspergillus* spp. to grow to detectable levels and dilute the concentration of DNA from nonviable organisms. To increase the sensitivity of qPCR methods, a larger sample volume for downstream DNA extraction is highly recommended (Sarma et al., 2020). Molecular methods, however, can lead to false positives because of the cross-reactivity with non-specified *Aspergillus* species. Since genetic materials from dead organisms are still detectable by qPCR after cannabis irradiation (Frink et al., 2022), assessment of the success of the decontamination process must not solely rely on qPCR and should include culture-based methods unless methods to circumvent these issues are used. These methods include the 48-h enrichment method described above and the use of nucleases that do not penetrate living cell walls or cell membranes but can degrade DNA of nonviable organisms. However, these methods have not been validated on cannabis treated with the decontamination methods currently used in cannabis production. Different forms of decontamination may require different enrichment and PCR techniques to perfectly match plating. Because decontamination techniques have not been certified by AOAC for hemp, no PCR kits are certified to work with decontaminated products. Irradiated products will require enrichments methods similar to those described above for molecular methods to identify only living organisms.

### 5.3. Methods for assessment of mycotoxins

Analytical methods for testing mycotoxin must be accurate, reproducible, and sensitive, but considerations such as speed and cost are also important if the method is to be adopted for routine testing. In cannabis, the Association of Official Analytical Collaboration (AOAC) International (2021) established target levels for limits of quantification (LOQ) for OTA, aflatoxin B_1_ and total aflatoxins (aflatoxin B_1_ + aflatoxin B_2_ + aflatoxin G_1_ + aflatoxin G_2_). In order to be accepted as an AOAC International method, LOQ must be below target levels; however, in some countries (e.g., EU and Australia) regulatory limits are much lower than the LOQs established by Association of Official Analytical Collaboration (AOAC) International (2021). Sample size and sample extraction methods play a pivotal role, and these methods vary for individual laboratories and technologies. Gas chromatography–mass spectrometry is used to monitor mycotoxins in other systems, but there have been no reports on its use in analysis of cannabis or hemp.

#### 5.3.1. Liquid chromatography

Liquid chromatography coupled with MS (LC–MS) is commonly used to detect and identify small molecules in complex matrices, such as cannabis and cannabis-infused products (Table 7). Tandem MS (LC–MS/MS) allows multiresidue methods that detect and quantify both mycotoxins and pesticide contaminants. Typically, these commercial methods target only those specified by AOAC International and does not target other mycotoxins discussed above; however, there are a few studies that have detected contaminants beyond those specified by the AOAC International. Five categories of bioactive compounds were identified (cannabinoids, primary metabolites, secondary metabolites, contaminants, extracted and leached compounds) using technology that coupled a Quick, Easy, Cheap, Effective, Rugged and Safe (QuEChERS) method for sample extraction and separation with identification by non-targeted LC–MS/MS. Contaminants identified by this method included a synthetic cannabinoid, nicotine, 11 drugs, 15 pesticides, and sterigmatocystin, the precursor to aflatoxin biosynthesis, but no aflatoxin was detected (Stempfer et al., 2021). Sixteen mycotoxins (aflatoxins, HT-2, T-2, α-and β-zearalenol, ZEA, beauvericin, and enniatins) were quantified in spiked samples using a QuEChERS method and quantified by ultra-high-performance LC coupled with quadrupole Orbitrap high-resolution MS, but only six mycotoxins were isolated from cannabis-based samples (Narváez et al., 2020; Table 6). To enhance detection, immunoaffinity columns with antibodies specific for aflatoxins and OTA have also been used for cannabis and hemp sample clean-up before LC analysis (Wilcox et al., 2020; Greaves et al., 2021; Buchicchio et al., 2022).

**Table 7:** Summary of advantages and disadvantages of methods reported for testing of mycotoxins in cannabis and hemp.

Analytical method	Advantages	Disadvantages
Liquid chromatography (LC) coupled with mass spectrometry LC–MS Targeted and nontargeted tandem MS (LC–MS/MS) Quadrupole Orbitrap high-resolution MS	Highly sensitive and efficient When coupled with MS, unknown compounds and minor components can be identified. Multiple compounds (e.g., cannabinoids, mycotoxins, and pesticides) can be detected in single assay	Extraction and analysis can be time-consuming Because cannabis is a complex matrix, method development can be difficult. Different extraction methods lead to different results QuEChERS x methods can increase extraction of mycotoxins Immunoaffinity columns can increase detection but are selective for specific mycotoxins Requires trained operators and expensive equipment Requires organic solvents
LC with fluorescence detection	Effective for specific known mycotoxins	Required immunoaffinity column for extracts prior to detection. Less sensitive than LC–MS Requires organic solvents
ELISA	Rapid, effective test for specific compounds Test kits available for other crops have been used for cannabis	No certified methods for cannabis Limited to antisera specific for toxins. To look at different mycotoxins, multiple tests must be performed.

#### 5.3.2. Immuno-based technologies

Analysis by LC is time-consuming and requires both trained operators and expensive equipment, so strategies that allow for simpler and more rapid detection of mycotoxins in food samples are desired (Xiong et al., 2022). The most commonly used of these methods is an enzyme-linked immunosorbent assay (ELISA); however, like the immunoaffinity columns above, ELISA is limited by the specificity and availability of mycotoxin-specific antibodies. ELISA is an excellent screening tool that can be used to estimate mycotoxins in other crops and cannabis plants (Bergstrom et al., 2019, 2020). Tests have been certified for rapid (≤30 min) determination of single mycotoxins (aflatoxin, DON, OTA, fumonisin, and ZEA) in grains [United States Federal Grain Inspection Services (FGIS), 2023], but there have been no certifications for use in cannabis or hemp. Dipsticks are rapid tests that can be performed rapidly (usually providing results in 30 min). Most dipstick tests are immuno-based assays that have antibodies immobilized on an analytical membrane and are used to verify mycotoxin content in food and grain. Dipstick tests can detect mycotoxins, either single or a group of compounds, and when portable photometric strip readers are used can support on-site screening (Lattanzio and Nivarlet, 2017). Gold nanoparticles (AuNPs) can be functionalized to recognize and bind to specific target molecules (Ferrari, 2023) and have been used as optical mycotoxin biosensors. A multiplex dipstick test using AuNP technology for detection of *Fusarium* mycotoxins in oats identified levels of multiple mycotoxins in a rapid on-site protocol, but this technique identified only relative levels of the toxins (e.g., negative, low positive, or positive; Lattanzio and Nivarlet, 2017). AuNPs are routinely used for detection in ELISA testing (Ferrari, 2023).

#### 5.3.3. Other emerging technologies

A DNA-based dipstick technology was developed to identify *Fusarium* spp. and trichothecene production potential” (Suga et al., 2022). E-nose technology is a rapid and cost-effective diagnostic tool for mycotoxin detection, but development of a universal e-nose is “unrealistic [because] a unique e-nose must be validated for each mycotoxin” (Cheli et al., 2023). In flow-injection analysis mass spectrometry (FIA-MS) chromatographic, chromatography separation steps are omitted. When FIA is coupled with triple quadrupole instruments and high-resolution mass spectrometers, such as time-of-flight (TOF) and Orbitrap instruments, high-throughput quantitative screening of analytes is possible. Rakk et al. (2023) validated FIA-MS methods that provided quantitative analysis of corn and wheat samples for 11 mycotoxins (aflatoxins, DON, fumonisins, HT-2, T-2, OTA, and ZEA) in less than 1 min.

## 6. Approaches to reduce fungi and mycotoxins on cannabis and hemp

The fungal and yeast species that have been found on cannabis and hemp can be reduced through various practices intended to minimize the incidence of propagules (mostly consisting of spores and/or mycelium) on the tissues or by reducing development under conditions favoring their spread. These practices can be grouped into pre-harvest and post-harvest management and are discussed in more detail below.

### 6.1. Pre-harvest management

Most strategies aimed at reducing fungal development on inflorescences before harvest are targeted to (i) reduce spread of fungal spores onto inflorescences, (ii) alter environmental conditions by reducing relative humidity in the growing environment, or (iii) prevent spread of spores by workers tending to the plants. For example, enhancing air circulation using fans in the final weeks leading up to harvest significantly reduced the numbers of total yeast and molds in cannabis inflorescences (*p* = 0.05; Punja et al., 2023). Genotypes of cannabis under cultivation can also affect the levels of fungi found to be present within the inflorescences and thus influence final quality of the product (Punja et al., 2023). Additional research is needed to develop genotypes that would allow cannabis producers to select plants that are less prone to high yeast and mold levels.

Based on observations of significant differences between *C. sativa* genotypes these differences may be based on chemical interactions with the fungal populations, but more knowledge is needed to determine if naturally occurring chemical compounds in cannabis inflorescences affect fungal development. This knowledge would allow breeders to target chemical profiles for the selection of new chemotypes. Although biological activities have been shown for extracts of *C. sativa* (summarized by Hourfane et al., 2023), few studies have tested activity against toxigenic fungi. Acetone extracts from *C. sativa* inflorescences and hashish inhibited both growth of *A. flavus* and the production of aflatoxin B1 but extracts of leaves and stems did not inhibit the fungus in poisoned food assays (Al Khoury et al., 2021). However, in disk diffusion and agar well diffusion assays, growth of *Fusarium* spp. and *A. niger* was inhibited by acetone, chloroform, ethanol, and water extracts of *C. sativa* leaves (Anjum and Zel-E-Arooj, 2018). For *A. niger*, inhibition zone in disk diffusion assays ranged from 20.6 mm (chloroform) to 23 mm (ethanol), and for *Fusarium* spp., inhibition zones ranged from 18.3 mm (chloroform) to 24.3 mm (aqueous). Hot water extracts of callus (CE) derived from leaves that contained alkaloids, terpenoids, and flavonoids, were active against *A. flavus*, *A. fumigatus*, *A. niger*, *F. solani*, and *Mucor* in an agar diffusion test. Inhibition zones (IZs) for CE treatments were greatest for *A. fumigatus* (15 mm) and IZs increased to 20 mm when the fungus was treated with zinc nanoparticles made from CE (CE-ZnONPs); the activity of CE-ZnONPs was greatest against *Mucor* (IZ = 30 mm), but the fungus was only slightly inhibited by of CE (IZ = 7 mm; Zaka et al., 2021). Growth of *F. oxysporum* was inhibited (up to 47%) by water extracts of *C. sativa* leaves (Tapwal et al., 2011). In greenhouse studies, nanoemulsions of the terpene-rich *C. sativa* by-products from commercial CBD extraction controlled powdery mildew diseases of hemp (Akinrinlola et al., 2022; Fei et al., 2023). These observations indicate that there are opportunities to enhance naturally occurring anti-fungal compounds in *C. sativa* to reduce levels of TYM.

Another area of research that can reduce potential levels of fungal contaminants is monitoring disease incidence and severity on cannabis and hemp inflorescences as they approach harvest and developing strategies that can reduce their levels pre-harvest. Such remediation approaches reduce the burden for post-harvest interventions that can alter the chemical composition and aroma profile (primarily a function of terpene composition and concentration) of the product. Application of registered microbial products to manage fungal pathogens infecting cannabis inflorescences should be undertaken with the knowledge that they may inadvertently increase total yeast and mold levels (Punja and Ni, 2022; Punja et al., 2023).

### 6.2. Post-harvest management

After harvesting, the trimming method used can influence the buildup of total yeast and molds in cannabis inflorescences (Punja et al., 2019). Furthermore, duration of drying and final moisture content (water activity) has a significant impact on the levels of fungi present, particularly those like *Aspergillus* and *Penicillium* spp. that are tolerant of low moisture levels (Punja et al., 2023). Storage method and duration and temperature can also influence the extent to which the dried inflorescences may build up yeasts and molds. Various post-harvest treatment methods can impact quality of cannabis-derived products with regard to yeast and mold levels. The use of irradiation by e-beam (ß-irradiation) or γ-irradiation is permitted in specific countries such as Canada and Israel and reduces fungal contaminants to zero (Punja, 2021d). These technologies are expensive, and although γ-irradiation did not change terpene profile in cannabis, treatment resulted in overall reduced levels of terpenes (Hazekamp, 2016). In medicinal cannabis, TYM levels of cannabis were reduced (6–4.5 log) when treated with γ-irradiation and by 5-log when treated with cold plasma treatment or e-beam (Jerushalmi et al., 2020). The relative advantages and disadvantages of other methods used for decontamination or sterilization in the food industry [e.g., heat; high pressure; chemical, filtration; extraction; non-ionizing and ionizing irradiation; photonic decontamination (i.e., X-ray); and cold plasma] for use in cannabis industries have been reviewed by Dhillon et al. (2022). In cases where cannabis use is destined for medical use by patients with immunocompromising conditions (e.g., cancer, AIDS, and diabetes), the use of these forms of irradiation may be worthy of consideration to ensure that fungal contaminants are reduced to negligible levels. The enhanced safety of these products should correspondingly reduce the incidence of various forms of fungal infections as reported in Section 3.

### 6.3. Reducing mycotoxins in cannabis tissues

After mycotoxins are produced either in the intact plant or the harvested inflorescences, there are no proven technologies to degrade or detoxify them, but cold plasma treatments have been reported to degrade mycotoxins in other systems (Doshi and Šerá, 2023); hence, cannabis products that have been sterilized or produced from sterilized materials may still contain mycotoxins. Byproducts formed by alteration of mycotoxins by varied mechanisms [opening of ring structure (aflatoxin B1, DON), closure of a ring structure (OTA), and oxidation, hydroxylation, and methylation (ZEA, FUM B1)] are usually but not always less harmful. A notable exception is the formation 𝛼-zearalanone, a more toxic compound, from ZEA (Liu et al., 2020). Flavonoids and phenolics in water extracts of plants have been implicated as active degrading agents of aflatoxins, but it is likely that higher molecular weight compounds, possibly enzymes, are involved (Loi et al., 2020). Essential oils of plants that contain fungistatic or fungicidal terpenes can potentially reduce mycotoxin content in cannabis products (Ranjith et al., 2021). For example, when the essential oil of *Heliotropium bacciferum* containing 32.9% a-pinene and 9.4% β-myrcene was mixed with aflatoxin B1, 82.6% of the mycotoxin was degraded (Al-Harrasi et al., 2021). Biocontrol agents may also affect mycotoxin levels. In co-culture studies, some antagonistic *Trichoderma* strains were able to glycosylate DON or convert ZEA into its hydroxylated derivative, β-zearalenol (Modrzewska et al., 2022). Therefore, there are opportunities for further research into decontamination of cannabis or hemp products should *Fusarium* mycotoxins become a regulatory issue.

## 7. Future directions for research

Current knowledge of the extent of fungal contamination on cannabis and hemp inflorescences relies on publicly available data from published independent studies and cannabis compliance tests. The latter is only available in a few legalized jurisdictions where such data are publicly disclosed [California Bureau of Cannabis Control (CBCC), 2018; Li et al., 2023]. Therefore, essential information on the incidence and frequency of several fungal species of concern is unavailable. In addition, most compliance tests currently rely on culture-based isolation (e.g., TYM) that provide no information on which specific fungal contaminants are present. While aflatoxins and OTA are regulated in many legalized jurisdictions, there are no US states or any country that currently regulate *Fusarium* spp. and their mycotoxins. As such, the incidence of *Fusarium* contamination is currently unknown in cannabis and hemp products, in contrast to cereal grains and many other agricultural commodities worldwide. In a recent survey of diagnostic professionals in the United States, infection by *Fusarium* spp. on cannabis or hemp in most areas of the country, except for the Great Plains region (Munir et al., 2023); this is likely due to the low numbers of samples received from this region. Similarly Canadian production areas have reported f *Fusarium* occurrence on cannabis plants and inflorescences during commercial cultivation (Punja et al., 2019, 2021, 2023; Punja, 2021a,b,c; Punja and Ni, 2022). Continued surveillance for the prevalence of *Fusarium* species and other phytopathogens that could impact the health of cannabis consumers, particularly those with immunocompromised systems, should be undertaken in production areas annually.

While LC–MS–MS methods are available to detect multiple mycotoxins together with pesticide residues in cannabis and hemp flowers (see Section 5.3), most methods used in cannabis or hemp compliance laboratories are largely based on existing regulatory action levels. Hence, *Fusarium* mycotoxins are not being included in compliance testing. Further research is needed to develop commercial analytical methods that would allow data collection to determine to what extent these potentially widespread but unregulated mycotoxins occur in cannabis and hemp products. In addition, large-scale international and national collaborations are needed to collect and disseminate information on fungal contaminants found in cannabis and hemp, similar to existing efforts in other agricultural commodities (e.g., NFCFD) for surveillance purposes. These data should be made available in the peer-reviewed literature.

## 8. Conclusion

A review of the current literature indicates there are potential human health risks of fungal and mycotoxin contaminants in cannabis and hemp flowers, particularly for the subset of the population that is immunocompromised. Further research is needed to understand contamination prevalence, inform regulatory policies, and advance management practices. Several studies have linked cannabis use to increased risk of fungal infection in immunocompromised patients. Current regulations have addressed the potential for harm from *Aspergillus* species, yet few reports have confirmed whether mycotoxin production is widespread or is rare in occurrence in cannabis products. While there are many reports of cannabis and hemp diseases caused by *Fusarium* species, there are no regulations currently in place for assessing mycotoxin levels. Additional research is needed to survey the extent of both *Aspergillus* and *Fusarium* species occurrence and potential mycotoxin presence.

A major hurdle faced by cannabis and hemp industries is addressing the disconnect between production-related issues and human safety issues. The current regulatory requirement for enumerating TYM present in cannabis and hemp tissues using plating on culture media does not allow for a direct interpretation as to potential health risks to consumers. The huge variation between United States jurisdictions in the acceptable levels of TYM, ranging from 100 to 100,000 cfu/g illustrates the difficulty in implementation of human health risk assessment. A consistent and widely adopted protocol for sampling, analysis, and interpretation of TYM results is essential to provide a consistent quality assessment for consumers and health practitioners. We suggest consideration of exclusion of non-harmful fungal and yeast species from *a priori* testing and instead encourage the adoption of species testing targeting a smaller range of fungi with potential to cause harm. The current reporting of “everything that is present” in cannabis samples may not serve the cannabis industry well moving forward since other potential harmful fungi such as *Fusarium* and *Penicillium* can be under-represented in the overall TYM counts. We recommend testing for the presence of *Aspergillus*, *Penicillium*, *Fusarium*, and *Mucor* as priorities, with an adjustment to adding other species if they are discovered to have potential harm. Additionally, multi-residue analytical methods (i.e., LC–MS–MS) used by most compliance testing laboratories should be further developed to enable the detection of *Fusarium* mycotoxins together with regulated contaminants.

One possible solution to reduce potential harm to medical users of cannabis from toxigenic fungi is to develop a two-tier system that distinguishes products intended for medical and recreational use. The products for medical use should have a unified national standard that includes mandatory irradiation to eliminate fungal contaminants and protect immunocompromised patients. The products for recreational use would have a higher tolerance for TYM (e.g., 50,000 cfu/g). The latter would still require mandatory testing for the presence of *Aspergillus* and *Fusarium* to provide assurances to consumers that they pose no harm from fungal contaminants. A zero-tolerance policy seems untenable for a plant-derived product exposed to many variables in the cultivation environment. There are many fungal species present in cannabis inflorescences that are not deemed to be of concern to human health. Rather, regulatory agencies could impose a limit of 10 cfu/g for species of concern such as *Aspergillus* and *Fusarium*, which may be sufficient to provide assurances to consumers while reducing the burden on an industry that already experiences significant regulation.

## Author contributions

KG: Conceptualization, Data curation, Investigation, Project administration, Visualization, Writing – original draft, Writing – review & editing. ML: Conceptualization, Investigation, Resources, Supervision, Writing – original draft, Writing – review & editing. AS: Investigation, Resources, Writing – review & editing. ZP: Conceptualization, Resources, Writing – original draft, Writing – review & editing.

## References

[ref1] Abrams D. I. (2022). Cannabis, cannabinoids, and cannabis-based medicines in cancer care. Integr. Cancer Ther. 21:15347354221081772. doi: 10.1177/15347354221081772 35225051 PMC8882944

[ref2] Akinrinlola R. J. Gwinn K. D. Wang T. Hansen Z. R. (2022). Numerous fungicides suppress hemp powdery mildew in the greenhouse in Tennessee, 2021. Available at: https://www.plantmanagementnetwork-org.utk.idm.oclc.org/pub/trial/PDMR/reports/2022/V049.pdf (Accessed August 2, 2023).

[ref3] Al Khoury A. Sleiman R. Atoui A. Hindieh P. Maroun R. G. Bailly J. D.. (2021). Antifungal and anti-aflatoxigenic properties of organs of *Cannabis sativa* L.: relation to phenolic content and antioxidant capacities. Arch. Microbiol. 203, 4485–4492. doi: 10.1007/s00203-021-02444-x 34143269

[ref4] Alam B. Lî J. Gě Q. Khan M. A. Gōng J. Mehmood S.. (2021). Endophytic fungi: from symbiosis to secondary metabolite communications or vice versa? Front. Plant Sci. 12:791033. doi: 10.3389/fpls.2021.791033 34975976 PMC8718612

[ref5] Alapont C. López-Mendoza M. C. Gil J. V. Martínez-Culebras P. V. (2014). Mycobiota and toxigenic *Penicillium* species on two Spanish dry-cured ham manufacturing plants. Food Addit. Contam. A Chem. Anal. Control Expo. Risk Assess. 31, 93–104. doi: 10.1080/19440049.2013.849007 24279369

[ref6] Alhamad T. Koraishy F. M. Lam N. N. Katari S. Naik A. S. Schnitzler M. A.. (2019). Cannabis dependence or abuse in kidney transplantation: implications for posttransplant outcomes. Transplantation 103, 2373–2382. doi: 10.1097/TP.0000000000002599 30747847 PMC6679817

[ref7] Al-Harrasi M. M. A. Al-Sadi A. M. Al-Sabahi J. N. Al-Farsi K. Waly M. I. Velazhahan R. (2021). Essential oils of *Heliotropium bacciferum, Ocimum dhofarense* and *Zataria multiflora* exhibit aflatoxin B1 detoxification potential. All Life 14, 989–996. doi: 10.1080/26895293.2021.1991006

[ref8] Altibi A. M. Seth R. Battisha A. Kak V. (2020). Cutaneous fusariosis in a patient with Job’s (hyper-IgE) syndrome. Case Rep. Infect. Dis. 2020:3091806. doi: 10.1155/2020/3091806 32607263 PMC7315260

[ref9] Anjum M. Zel-E-Arooj A.S., Rehman P., and Khadim J. (2018). Evaluation of antimicrobial activity and ethnobotanical study of *Cannabis sativa* L. Pure Appl. Biol. 7, 706–713. doi: 10.19045/bspab.2018.70088

[ref10] Assaf C. E. H. Zetina-Serrano C. Tahtah N. Khoury A. E. Atoui A. Oswald I. P.. (2020). Regulation of secondary metabolism in the *Penicillium* genus. Int. J. Mol. Sci. 21:9462. doi: 10.3390/ijms21249462 33322713 PMC7763326

[ref11] Association of Official Analytical Collaboration (AOAC) International (2021). Standard method performance requirements (SMPRs®) for quantitative analysis of mycotoxins in cannabis biomass and cannabis-derived products. Available at: https://www.aoac.org/wp-content/uploads/2021/12/SMPR-2021_010.pdf (Accessed August 2, 2023).

[ref12] Ball O. J. -P. Coudron T. A. Tapper B. A. Davies E. Trently D. J. Bush L. P.. (2006). Importance of host plant species, *Neotyphodium* endophyte isolate, and alkaloids on feeding by *Spodoptera frugiperda* (Lepidoptera: Noctuidae) larvae. J. Econ. Entomol. 99, 1462–1473. doi: 10.1093/jee/99.4.1462, PMID: 16937705

[ref13] Ball O. J. -P. Gwinn K. D. Pless C. D. Popay A. J. (2011). Endophyte isolate and host grass effects on *Chaetocnema pulicaria* (Coleoptera: Chrysomelidae) feeding. J. Econ. Entomol. 104, 665–672. doi: 10.1603/EC10262 21510220

[ref14] Banani H. Marcet-Houben M. Ballester A. R. Abbruscato P. González-Candelas L. Gabaldón T.. (2016). Genome sequencing and secondary metabolism of the postharvest pathogen *Penicillium griseofulvum*. BMC Genomics 17:19. doi: 10.1186/s12864-015-2347-x, PMID: 26729047 PMC4700700

[ref15] Barkai-Golan R. (2008). “*Penicillium* mycotoxins” in Mycotoxins in Fruits and Vegetables. eds. Barkai R. Paster N. (San Diego, CA: Academic Press), 153–183.

[ref16] Barnett S. E. Cala A. R. Hansen J. L. Crawford J. Viands D. R. Smart L. B.. (2020). Evaluating the microbiome of hemp. Phytobiomes J. 4, 351–363. doi: 10.1094/PBIOMES-06-20-0046-R

[ref17] Benedict K. Thompson G. R. 3rd Jackson B. R. (2020). Cannabis use and fungal infections in a commercially insured population, United States, 2016. Emerg. Infect. Dis. 26, 1308–1310. doi: 10.3201/eid2606.191570 32441624 PMC7258471

[ref18] Bergstrom G. Starr J. Myers K. (2020). Diseases affecting hemp in New York. Cornell Hemp. Available at: https://bpb-us-e1.wpmucdn.com/blogs.cornell.edu/dist/a/7491/files/2019/08/2019-Hemp-Diseases-Field-Day-Handout8.9.19.pdf (Accessed August 2, 2023).

[ref19] Bergstrom G. Starr J. Myers K. Cummings J. (2019). “An early view of diseases affecting hemp in New York” in Science of hemp: Production and management (University of Kentucky College of agriculture food and environment SR-112). Available at: https://plantpathology.ca.uky.edu/files/sr112.pdf (Accessed October 11, 2021).

[ref20] Bokhari F. Aly M. M. (2009). Patulin production by *Penicillium glabrum* isolated from *Coffea arabica* L. and the activities of some natural antifungal and antimycotoxin plants. Egypt. J. Microbiol. 44, 47–59. doi: 10.21608/EJM.2009.285

[ref21] Bonnet M. S. Roux J. Mounien L. Dallaporta M. Troadec J. D. (2012). Advances in deoxynivalenol toxicity mechanisms: the brain as a target. Toxins 4, 1120–1138. doi: 10.3390/toxins4111120 23202308 PMC3509700

[ref22] Boudra H. Le Bars P. Le Bars J. (1995). Thermostability of ochratoxin a in wheat under two moisture conditions. Appl. Environ. Microbiol. 61, 1156–1158. doi: 10.1128/aem.61.3.1156-1158.1995 7793917 PMC167371

[ref23] Brandhorst T. Dowd P. F. Kenealy W. R. (1996). The ribosome-inactivating protein restrictocin deters insect feeding on *Aspergillus restrictus*. Microbiology 142, 1551–1556. doi: 10.1099/13500872-142-6-1551 8704996

[ref24] Bräse S. Encinas A. Keck J. Nising C. F. (2009). Chemistry and biology of mycotoxins and related fungal metabolites. Chem. Rev. 109, 3903–3990. doi: 10.1021/cr050001f 19534495

[ref25] Bretz M. Beyer M. Cramer B. Knecht A. Humpf H. U. (2006). Thermal degradation of the fusarium mycotoxin deoxynivalenol. J. Agric. Food Chem. 54, 6445–6451. 16910743 10.1021/jf061008g

[ref26] Buchicchio L. Asselborn L. Schneider S. van Nieuwenhuyse A. Moris G. Schummer C. (2022). Investigation of aflatoxin and ochratoxin a contamination of seized cannabis and cannabis resin samples. Mycotoxin Res. 38, 71–78. doi: 10.1007/s12550-022-00449-z 35028912

[ref27] Bui-Klimke T. R. Wu F. (2015). Ochratoxin a and human health risk: a review of the evidence. Crit. Rev. Food Sci. Nutr. 55, 1860–1869. doi: 10.1080/10408398.2012.724480 24874522 PMC4247821

[ref28] Bulgari D. Fiorini L. Gianoncelli A. Bertuzzi M. Gobbi E. (2020). Enlightening gliotoxin biological system in agriculturally relevant *Trichoderma* spp. Front. Microbiol. 11:200. doi: 10.3389/fmicb.2020.00200 32226413 PMC7080844

[ref29] Cai J. Yan R. Shi J. Chen J. Long M. Wu W.. (2022). Antifungal and mycotoxin detoxification ability of essential oils: a review. Phytother. Res. 36, 62–72. doi: 10.1002/ptr.7281 34528300

[ref30] California Bureau of Cannabis Control (CBCC) (2018). Bureau of cannabis control announces weekly reports. Available at: https://cannabis.ca.gov/2018/10/bureau-of-cannabis-control-announces-weekly-reports/ (Accessed August 7, 2023).

[ref31] Caplan D. Matzneller P. Gutierrez D. (2022). “Harvest and post-harvest” in Handbook of Cannabis Production in Controlled Environments. ed. Zheng Y. (Boca Raton, FL: CRC Press), 291–310.

[ref32] Carter B. P. (2019). The what, why and how of water activity in cannabis. Cannabis Sci. Tech. 2, 30–35.

[ref33] Cescon D. W. Page A. V. Richardson S. Moore M. J. Boerner S. Gold W. L. (2008). Invasive pulmonary aspergillosis associated with marijuana use in a man with colorectal cancer. J. Clin. Oncol. 26, 2214–2215. doi: 10.1200/JCO.2007.15.2777 18445848

[ref34] Chacon F. T. Raup-Konsavage W. M. Vrana K. E. Kellogg J. J. (2022). Secondary terpenes in *Cannabis sativa* L.: synthesis and synergy. Biomedicine 10:3142. doi: 10.3390/biomedicines10123142 PMC9775512 36551898

[ref35] Cheli F. Ottoboni M. Fumagalli F. Mazzoleni S. Ferrari L. Pinotti L. (2023). E-nose technology for mycotoxin detection in feed: ready for a real context in field application or still an emerging technology? Toxins 15:146. doi: 10.3390/toxins15020146 36828460 PMC9958648

[ref36] Chen J. Chen Y. Zhu Q. Wan J. (2023). Ochratoxin a contamination and related high-yield toxin strains in Guizhou dried red chilies. Food Control 145:109438. doi: 10.1016/j.foodcont.2022.109438

[ref37] Chen S. C. A. Meyer W. Sorrell T. C. Halliday C. L. Landry M. L. McAdam A. J.. (2019). Manual of Clinical Microbiology 12th Edn. Washington, DC: ASM Press

[ref38] Chih S. McDonald M. Dipchand A. Kim D. Ducharme A. Kaan A.. (2020). Canadian cardiovascular society/Canadian cardiac transplant network position statement on heart transplantation: patient eligibility, selection, and post-transplantation care. Can. J. Cardiol. 36, 335–356. doi: 10.1016/j.cjca.2019.12.025 32145863

[ref39] Chiminelli I. Spicer L. J. Maylem E. R. S. Caloni F. (2022). Emerging mycotoxins and reproductive effects in animals: a short review. J. Appl. Toxicol. 42, 1901–1909. doi: 10.1002/jat.4311 35229323

[ref40] Choi B. K. Cho J. H. Jeong S. H. Shin H. S. Son S. W. Yeo Y. K.. (2012). Zearalenone affects immune-related parameters in lymphoid organs and serum of rats vaccinated with porcine parvovirus vaccine. Toxicol. Res. 28, 279–288. doi: 10.5487/TR.2012.28.4.279 24278621 PMC3834426

[ref41] Comeau D. Novinscak A. Joly D. L. Filion M. (2020). Spatio-temporal and cultivar-dependent variations in the cannabis microbiome. Front. Microbiol. 11:491. doi: 10.3389/fmicb.2020.00491 32265895 PMC7105690

[ref42] da Silva-Filho F. de Souza M. Rezende G. da Silva F. da Cruz J. da Silva G.. (2021). Screening of alkaloid-producing endophytic *Penicillium* strains from amazon medicinal plants by electrospray ionization mass spectrometry (ESI-MS) and principal component analysis (PCA). J. Braz. Chem. Soc. 32, 1832–1839. doi: 10.21577/0103-5053.20210074

[ref43] Dai C. Chen C. Guan D. Chen H. Wang F. Wang W.. (2020). Pesimquinolones produced by *Penicillium simplicissimum* and their inhibitory activity on nitric oxide production. Phytochemistry 174:112327. doi: 10.1016/j.phytochem.2020.112327 32222549

[ref44] Dhillon S. G. Hukkeri S. Nightingale D. Callaway J. (2022). Evaluation of different techniques to decontaminate medical cannabis and their effect on cannabinoid content. ACS Agric. Sci. Technol. 2, 1126–1133. doi: 10.1021/acsagscitech.2c00231

[ref45] Díaz G. A. Torres R. Vega M. Latorre B. A. (2009). Ochratoxigenic *Aspergillus* species on grapes from Chilean vineyards and Aspergillus threshold levels on grapes. Int. J. Food Microbiol. 133, 195–199. doi: 10.1016/j.ijfoodmicro.2009.04.018 19464066

[ref510] dos Santos C. M. C. da Costa G. L. Figueroa-Villar J. D. (2012). Identification of citrinin as the defence metabolite of *Penicillium corylophilum* stressed with the antagonist fungus *Beauveria bassiana*. Nat. Prod. Res. 26:2316–2311. doi: 10.1080/14786419.2012.668690 22414191

[ref46] Doshi P. Šerá B. (2023). Role of non-thermal plasma in *Fusarium* inactivation and mycotoxin decontamination. Plan. Theory 12:627. doi: 10.3390/plants12030627 PMC9921801 36771708

[ref47] European Pharmacopoeia Commission (2023). European Pharmacopoeia 11th Edn. France: Council of Europe

[ref48] Eykelbosh A. (2021). Unregulated cannabis: risky production practices raise concern for consumers. Available at: https://ncceh.ca/content/blog/unregulated-cannabis-risky-production-practices-raise-concern-consumers (Accessed August 2, 2023).

[ref49] Ezekiel C. N. Oyedele O. A. Kraak B. Ayeni K. I. Sulyok M. Houbraken J.. (2020). Fungal diversity and mycotoxins in low moisture content ready-to-eat foods in Nigeria. Front. Microbiol. 11:615. doi: 10.3389/fmicb.2020.00615 32328050 PMC7161469

[ref50] Fehr K. O. Kalant H. (1972). Analysis of cannabis smoke obtained under different combustion conditions. Can. J. Physiol. Pharmacol. 50, 761–767. doi: 10.1139/y72-111 5053787

[ref51] Fei T. Gwinn K. D. Leyva-Gutierrez F. M. A. Wang T. (2023). Nanoemulsions of terpene by-products from cannabidiol production have promising insecticidal effect on *Callosobruchus maculatus*. Heliyon 9:e15101. doi: 10.1016/j.heliyon.2023.e15101 37095909 PMC10121836

[ref52] Feizollahi E. Roopesh M. S. (2022). Mechanisms of deoxynivalenol (DON) degradation during different treatments: a review. Crit. Rev. Food Sci. Nutr. 62, 5903–5924. doi: 10.1080/10408398.2021.1895056 33729830

[ref53] Ferrari E. (2023). Gold nanoparticle-based plasmonic biosensors. Bios 13:411. doi: 10.3390/bios13030411 PMC10046074 36979623

[ref54] Feys S. Lagrou K. Lauwers H. M. Haenen K. Jacobs C. Brusselmans M.. (2023). High burden of COVID-19-associated pulmonary aspergillosis (CAPA) in severely immunocompromised patients requiring mechanical ventilation. Clin. Infect. Dis. 11:ciad546. doi: 10.1093/cid/ciad546 PMC10874259 37691392

[ref55] Fogle M. R. Douglas D. R. Jumper C. A. Straus D. C. (2008). Growth and mycotoxin production by *Chaetomium globosum* is favored in a neutral pH. Int. J. Mol. Sci. 9, 2357–2365. doi: 10.3390/ijms9122357 19330080 PMC2635641

[ref56] Fraga M. E. Direito G. M. Gatti M. J. Moraes A. M. L. Cavaglieri L. R. Dalcero A. M. (2008). Revaluation of aflatoxin production by *Aspergillus candidus* and *Eurotium chevalieri* isolated from poultry feed in Brazil. Rev. Bras. Med. Vet. 30, 86–90.

[ref57] Frink S. Marjanovic O. Tran P. Wang Y. Guo W. Encarnacion N.. (2022). Use of X-ray irradiation for inactivation of *Aspergillus* in cannabis flower. PLoS One 17:e0277649. doi: 10.1371/journal.pone.0277649 36378669 PMC9665375

[ref58] Frisvad J. C. (2018). A critical review of producers of small lactone mycotoxins: patulin, penicillic acid and moniliformin. World Mycotoxin J. 11, 73–100. doi: 10.3920/WMJ2017.2294

[ref59] Frisvad J. C. Filtenborg O. (1990). “Revision of *Penicillium* subgenus *Furcatum* based on secondary metabolites and conventional characters” in Modern Concepts in *Penicillium* and *Aspergillus* Classification. eds. Samson R. A. Pitt J. I. (Boston, MA: Springer), 159–172.

[ref60] Frisvad J. C. Houbraken J. Popma S. Samson R. A. (2013). Two new *Penicillium species Penicillium buchwaldii* and *Penicillium spathulatum*, producing the anticancer compound asperphenamate. FEMS Microbiol. Lett. 339, 77–92. doi: 10.1111/1574-6968.12054 23173673

[ref61] Frisvad J. C. Hubka V. Ezekiel C. N. Hong S. B. Nováková A. Chen A. J.. (2019). Taxonomy of *Aspergillus* section *Flavi* and their production of aflatoxins, ochratoxins and other mycotoxins. Stud. Mycol. 93, 1–63. doi: 10.1016/j.simyco.2018.06.001 30108412 PMC6080641

[ref62] Frisvad J. C. Møller L. L. H. Larsen T. O. Kumar R. Arnau J. (2018). Safety of the fungal workhorses of industrial biotechnology: update on the mycotoxin and secondary metabolite potential of *Aspergillus niger, Aspergillus oryzae,* and *Trichoderma reesei*. Appl. Microbiol. Biotechnol. 102, 9481–9515. doi: 10.1007/s00253-018-9354-1 30293194 PMC6208954

[ref63] Gagnon M. McRitchie T. Montsion K. Tully J. Blais M. Snider N.. (2023). High levels of pesticides found in illicit cannabis inflorescence compared to licensed samples in Canadian study using expanded 327 pesticides multiresidue method. J. Cannabis Res. 5:34. doi: 10.1186/s42238-023-00200-0 37620969 PMC10463991

[ref64] Gao M. Glenn A. E. Gu X. Mitchell T. R. Satterlee T. Duke M. V.. (2020). Pyrrocidine, a molecular off switch for fumonisin biosynthesis. PLoS Pathog. 16:e1008595. doi: 10.1371/journal.ppat.1008595 32628727 PMC7377494

[ref65] Gargani Y. Bishop P. Denning D. W. (2011). Too many mouldy joints—marijuana and chronic pulmonary aspergillosis. Mediter. J. Hematol. Infect. Dis. 3:e2011005. doi: 10.4084/mjhid.2011.005 PMC3103256 21625309

[ref66] Gautam A. K. Kant M. Thakur Y. (2013). Isolation of endophytic fungi from *Cannabis sativa* and study their antifungal potential. Arch. Phytopathol. Pflanzenschutz. 46, 627–635. doi: 10.1080/03235408.2012.749696

[ref67] Gauthier N. W. Thiessen L. D. (Ed.). (2022). Compendium of Cannabis Diseases. Minnesota: APS Publications

[ref68] Gherbawy Y. Shebany Y. (2018). Mycobiota, total aflatoxins and ochratoxin a of cardamom pods. Food Sci. Technol. 24, 87–96. doi: 10.3136/fstr.24.87

[ref69] Gil-Serna J. Vázquez C. Patiño B. (2020). Genetic regulation of aflatoxin, ochratoxin a, trichothecene, and fumonisin biosynthesis: a review. Int. Microbiol. 23, 89–96. doi: 10.1007/s10123-019-00084-2 31144067

[ref70] Goldman S. Bramante J. Vrdoljak G. Guo W. Wang Y. Marjanoviccaps O.. (2021). The analytical landscape of cannabis compliance testing. J. Liq. Chromatogr. Relat. 44, 403–420. doi: 10.1080/10826076.2021.1996390

[ref71] Greaves A. Maddison K. Doran M. Lin S. Geiling B. (2021). Single-laboratory validation of an immunoaffinity column cleanup LC method for the analysis of aflatoxins and ochratoxin a in cannabis plant material, resins, vapes, isolates, and edible products. J. AOAC Int. 104, 1264–1271. doi: 10.1093/jaoacint/qsab057 33881521

[ref72] Gruber-Dorninger C. Novak B. Nagl V. Berthiller F. (2017). Emerging mycotoxins: beyond traditionally determined food contaminants. J. Agric. Food Chem. 65, 7052–7070. doi: 10.1021/acs.jafc.6b03413 27599910

[ref73] Gwinn K. D. Hansen Z. Kelly H. Ownley B. H. (2022). Diseases of *Cannabis sativa* caused by diverse *Fusarium* species. Front. Agron. 3:796062. doi: 10.3389/fagro.2021.796062

[ref74] Hareeri R. H. Aldurdunji M. M. Abdallah H. M. Alqarni A. A. Mohamed S. G. A. Mohamed G. A.. (2022). *Aspergillus ochraceus*: metabolites, bioactivities, biosynthesis, and biotechnological potential. Molecules 27:6759. doi: 10.3390/molecules27196759 36235292 PMC9572620

[ref75] Hazekamp A. (2016). Evaluating the effects of gamma-irradiation for decontamination of medicinal cannabis. Front. Pharmacol. 7:108. doi: 10.3389/fphar.2016.00108 27199751 PMC4847121

[ref76] Health Canada (2020). Food and drug regulations, Ottawa. Available at: https://laws-lois.justice.gc.ca/eng/regulations/c.r.c.,_c._870/index.html (Accessed August 2, 2023).

[ref77] Houbraken J. Kocsubé S. Visagie C. M. Yilmaz N. Wang X. C. Meijer M.. (2020). Classification of *Aspergillus, Penicillium, Talaromyces* and related genera (*Eurotiales*): an overview of families, genera, subgenera, sections, series and species. Stud. Mycol. 95, 5–169. doi: 10.1016/j.simyco.2020.05.002 32855739 PMC7426331

[ref78] Hourfane S. Mechqoq H. Bekkali A. Y. Rocha J. M. El Aouad N. (2023). A comprehensive review on *Cannabis sativa* ethnobotany, phytochemistry, molecular docking and biological activities. Plan. Theory 12:1245. doi: 10.3390/plants12061245 PMC10058143 36986932

[ref79] Ismaiel A. A. Papenbrock J. (2015). Mycotoxins: producing fungi and mechanisms of phytotoxicity. Agriculture 5, 492–537. doi: 10.3390/agriculture5030492

[ref80] Ismail A. M. Elshewy E. S. El-Ganainy S. M. Magistà D. Hamouda A. F. Alhudaib K. A.. (2023). Mycotoxins from tomato pathogenic *Alternaria alternata* and their combined cytotoxic effects on human cell lines and male albino rats. J. Fungi 9:282. doi: 10.3390/jof9030282 PMC10054162 36983450

[ref81] Israel Ministry of Health—Medical Cannabis Unit (IMC) (2017) SOP 152, IMC-GMP production of cannabis for medical use. Available at: https://www.gov.il/BlobFolder/policy/mmk152-2016/he/files_circulars_mmk_mmk152_2016.pdf (Accessed August 2, 2023).

[ref82] Jameson L. E. Conrow K. D. Pinkhasova D. V. Boulanger H. L. Ha H. Jourabchian N.. (2022). Comparison of state-level regulations for cannabis contaminants and implications for public health. Environ. Health Perspect. 130:97001. doi: 10.1289/EHP11206 36102653 PMC9472674

[ref83] Jaster-Keller J. Müller M. E. H. El-Khatib A. H. Lorenz N. Bahlmann A. Mülow-Stollin U.. (2023). Root uptake and metabolization of *Alternaria* toxins by winter wheat plants using a hydroponic system. Mycotoxin Res. 39, 109–126. doi: 10.1007/s12550-023-00477-3 36929507 PMC10181980

[ref84] Jerushalmi S. Maymon M. Dombrovsky A. Freeman S. (2020). Fungal pathogens affecting the production and quality of medical cannabis in Israel. Plan. Theory 9:882. doi: 10.3390/plants9070882 PMC7412049 32668702

[ref85] Ji F. He D. Olaniran A. O. Mokoena M. P. Xu J. Shi J. (2019). Occurrence, toxicity, production and detection of fusarium mycotoxin: a review. Food Prod. Process Nutr 1:6. doi: 10.1186/s43014-019-0007-2

[ref86] Jiang H. Zhong S. Schwarz P. Chen B. Rao J. (2023). Antifungal activity, mycotoxin inhibitory efficacy, and mode of action of hop essential oil nanoemulsion against fusarium graminearum. Food Chem. 400:134016. doi: 10.1016/j.foodchem.2022.134016 36084588

[ref87] Kabak B. (2009). The fate of mycotoxins during thermal food processing. J. Sci. Food Agric. 89, 549–554. doi: 10.1002/jsfa.3491

[ref88] Kagen S. L. Kurup V. P. Sohnle P. G. Fink J. N. (1983). Marijuana smoking and fungal sensitization. J. Allergy Clin. Immunol. 71, 389–393. doi: 10.1016/0091-6749(83)90067-2 6833678

[ref89] Kamle M. Mahato D. K. Gupta A. Pandhi S. Sharma N. Sharma B.. (2022). Citrinin mycotoxin contamination in food and feed: impact on agriculture, human health, and detection and management strategies. Toxins 14:85. doi: 10.3390/toxins14020085 35202113 PMC8874403

[ref91] Karpiński T. M. (2020). Essential oils of Lamiaceae family plants as antifungals. Biomol. Ther. 10:103. doi: 10.3390/biom10010103 PMC7023020 31936168

[ref92] Kim H. Y. Park H. M. Lee C. H. (2012). Mass spectrometry-based chemotaxonomic classification of *Penicillium species* (*P. echinulatum*, *P. expansum*, *P. solitum*, and *P. oxalicum*) and its correlation with antioxidant activity. J. Microbiol. Methods 90, 327–335. doi: 10.1016/j.mimet.2012.06.006 22732319

[ref93] Kolawole O. Meneely J. Petchkongkaew A. Elliott C. (2021). A review of mycotoxin biosynthetic pathways: associated genes and their expressions under the influence of climatic factors. Fungal Biol. Rev. 37, 8–26. doi: 10.1016/j.fbr.2021.04.003

[ref94] Kouevidjin G. Mazieres J. Fayas S. Didier A. (2003). Aspergillose bronchopulmonaire allergique aggravée par la consommation de cannabis. Rev. Francaise D. Allergol. Et D. Immunol. Clin. 43, 192–194. doi: 10.1016/S0335-7457(03)00050-9

[ref95] Krane K. (2020). Cannabis legalization is key to economic recovery, much like ending alcohol prohibition helped us out of the great depression. Forbe. Available at: https://www.forbes.com/sites/kriskrane/2020/05/26/cannabis-legalization-is-key-to-economic-recovery-much-like-ending-alcohol-prohibition-helped-us-out-of-the-great-depression/?sh=617cd2ad3241 (Accessed August 3, 2023).

[ref96] Krawczeniuk A. S. O’Rear C. E. Mislivec P. B. Bruce V. R. Trucksess M. W. Llewellyn G. C. (1987). “Evaluating illicit marihuana for aflatoxins and toxigenic fungi” in Biodeterioration Research 1. eds. Llewellyn G. C. O’Rear C. E. (Boston, MA: Springer), 149–164.

[ref97] Kristy B. Carrell A. A. Johnston E. Klingeman D. Gwinn K. D. Syring K. C.. (2022). Chronic drought differentially alters the belowground microbiome of drought tolerant and drought susceptible genotypes of *Populus trichocarpa*. Phytobiomes 6, 317–330. doi: 10.1094/PBIOMES-12-21-0076-R

[ref98] Kuehn B. M. (2021). Aspergillosis is common among COVID-19 patients in the ICU. JAMA 326:1573. doi: 10.1001/jama.2021.17973 34698776

[ref99] Kurup V. P. Resnick A. Kagen S. L. Cohen S. H. Fink J. N. (1983). Allergenic fungi and actinomycetes in smoking materials and their health implications. Mycopathologia 82, 61–64. doi: 10.1007/BF00436948 6348548

[ref100] Kusari P. Kusari S. Spiteller M. Kayser O. (2013). Endophytic fungi harbored in *Cannabis sativa* L.: diversity and potential as biocontrol agents against host plant-specific phytopathogens. Fungal Divers. 60, 137–151. doi: 10.1007/s13225-012-0216-3

[ref101] Lass-Flörl C. Dietl A. M. Kontoyiannis D. P. Brock M. (2021). *Aspergillus terreus* species complex. Clin. Microbiol. Rev. 34:e0031120. doi: 10.1128/CMR.00311-20 34190571 PMC8404697

[ref102] Lattanzio V. M. Nivarlet N. (2017). Multiplex dipstick immunoassay for semiquantitative determination of fusarium mycotoxins in oat. Methods Mol. Biol. 1536, 137–142. doi: 10.1007/978-1-4939-6682-0_10 28132148

[ref103] Laut S. Poapolathep S. Piasai O. Sommai S. Boonyuen N. Giorgi M.. (2023). Storage fungi and mycotoxins associated with rice samples commercialized in Thailand. Foods 12:487. doi: 10.3390/foods12030487 36766016 PMC9914209

[ref104] Levi M. E. Montague B. T. Thurstone C. Kumar D. Huprikar S. S. Kotton C. N. (2019). Marijuana use in transplantation: a call for clarity. Clin. Transpl. 33:e13456. doi: 10.1111/ctr.13456 30506888

[ref105] Li B. Chen Y. Zhang Z. Qin G. Chen T. Tian S. (2020). Molecular basis and regulation of pathogenicity and patulin biosynthesis in *Penicillium expansum*. Compr. Rev. Food Sci. Food Saf. 19, 3416–3438. doi: 10.1111/1541-4337.12612 33337032

[ref106] Li L. Chmura S. E. Judd C. D. Duffy B. C. (2023). “New York perspectives of medical cannabis laboratory analysis” in Medicinal Usage of Cannabis and Cannabinoids. eds. Preedy V. R. Patel V. B. Martin C. R. (New York: Academic Press), 77–89.

[ref107] Lin K. Chang J. Y. Zou G. (2022). A case of COVID-19-associated pulmonary aspergillosis. Chest 162, A585–A586. doi: 10.1016/j.chest.2022.08.456

[ref108] Liu Y. Galani Yamdeu J. H. Gong Y. Y. Orfila C. (2020). A review of postharvest approaches to reduce fungal and mycotoxin contamination of foods. Compr. Rev. Food Sci. Food Saf. 19, 1521–1560. doi: 10.1111/1541-4337.12562 33337083

[ref109] Liu Y. Li M. Bian K. Guan E. Liu Y. Lu Y. (2019). Reduction of deoxynivalenol in wheat with superheated steam and its effects on wheat quality. Toxins 11:414. doi: 10.3390/toxins11070414 31315243 PMC6669746

[ref110] Llewellyn G. C. O'Rear C. E. (1977). Examination of fungal growth and aflatoxin production on marihuana. Mycopathologia 62, 109–112. doi: 10.1007/BF01259400 414138

[ref111] Loi M. Paciolla C. Logrieco A. F. Mulè G. (2020). Plant bioactive compounds in pre-and postharvest management for aflatoxins reduction. Front. Microbiol. 11:243. doi: 10.3389/fmicb.2020.00243 32226415 PMC7080658

[ref112] Mahmoudian F. Sharifirad A. Yakhchali B. Ansari S. Fatemi S. S. (2021). Production of mycophenolic acid by a newly isolated indigenous *Penicillium glabrum*. Curr. Microbiol. 78, 2420–2428. doi: 10.1007/s00284-021-02509-6 34019120 PMC8138112

[ref113] Malmstrøm J. Christophersen C. Frisvad J. C. (2000). Secondary metabolites characteristic of *Penicillium citrinum*, *Penicillium steckii* and related species. Phytochemistry 54, 301–309. doi: 10.1016/s0031-9422(00)00106-0 10870185

[ref114] Martyny J. W. Serrano K. A. Schaeffer J. W. Van Dyke M. V. (2013). Potential exposures associated with indoor marijuana growing operations. J. Occup. Environ. Hyg. 10, 622–639. doi: 10.1080/15459624.2013.831986 24116667

[ref115] McKernan K. Spangler J. Zhang L. Tadigotla V. Helbert Y. Foss T.. (2015). Cannabis microbiome sequencing reveals several mycotoxic fungi native to dispensary grade cannabis flowers. F1000Res 4:1422. doi: 10.12688/f1000research.7507.2 27303623 PMC4897766

[ref117] Micheluz A. Sulyok M. Manente S. Krska R. Varese G. C. Ravgnan G. (2016). Fungal secondary metabolite analysis applied to cultural heritage: the case of a contaminated library in Venice. World Mycotoxin J. 9, 397–407. doi: 10.3920/WMJ2015.1958

[ref118] Milani J. M. (2013). Ecological conditions affecting mycotoxin production in cereals: a review. Vet. Med. 58, 405–411. doi: 10.17221/6979-VETMED

[ref119] Min J. Y. Min K. B. (2018). Marijuana use is associated with hypersensitivity to multiple allergens in US adults. Drug Alcohol Depend. 182, 74–77. doi: 10.1016/j.drugalcdep.2017.09.039 29172121

[ref120] Mishra S. Priyanka Sharma S. (2022). Metabolomic insights into endophyte-derived bioactive compounds. Front. Microbiol. 13:835931. doi: 10.3389/fmicb.2022.835931 35308367 PMC8926391

[ref121] Modrzewska M. Błaszczyk L. Stępień Ł. Urbaniak M. Waśkiewicz A. Yoshinari T.. (2022). *Trichoderma versus Fusarium*-inhibition of pathogen growth and mycotoxin biosynthesis. Molecules 27:8146. doi: 10.3390/molecules27238146 36500242 PMC9735881

[ref122] Munir M. Leonberger K. Kesheimer K. A. Bolt M. Zuefle M. Aronson E.. (2023). Occurrence and distribution of common diseases and pests of US cannabis: a survey. Plant Health Prog. doi: 10.1094/PHP-01-23-0004-S

[ref123] Nardo F. D. Cavalera S. Baggiani C. Chiarello M. Pazzi M. Anfossi L. (2020). Enzyme immunoassay for measuring aflatoxin B1 in legal cannabis. Toxins 12:265. doi: 10.3390/toxins12040265 32326118 PMC7232199

[ref124] Narváez A. Rodríguez-Carrasco Y. Castaldo L. Izzo L. Ritieni A. (2020). Ultra-high-performance liquid chromatography coupled with quadrupole orbitrap high-resolution mass spectrometry for multi-residue analysis of mycotoxins and pesticides in botanical nutraceuticals. Toxins 12:114. doi: 10.3390/toxins12020114 32059484 PMC7076805

[ref125] National Academies of Sciences, Engineering, and Medicine (NASEM) (2017). The Health Effects of Cannabis and Cannabinoids: The Current State of Evidence and Recommendations for Research. Washington, DC: The National Academies Press 28182367

[ref126] Navale V. Vamkudoth K. R. Ajmera S. Dhuri V. (2021). *Aspergillus* derived mycotoxins in food and the environment: prevalence, detection, and toxicity. Toxicol. Rep. 8, 1008–1030. doi: 10.1016/j.toxrep.2021.04.013 34408970 PMC8363598

[ref127] Nguyen L. C. Yang D. Nicolaescu V. Best T. J. Gula H. Saxena D.. (2022). Cannabidiol inhibits SARS-CoV-2 replication through induction of the host ER stress and innate immune responses. Sci. Adv. 8:eabi6110. doi: 10.1126/sciadv.abi6110 35050692 PMC11816653

[ref128] Oar M. A. Savage C. H. Rufer E. S. Rucker R. P. Guzman J. A. (2022). Thermography of cannabis extract vaporization cartridge heating coils in temperature and voltage-controlled systems during a simulated human puff. PLoS One 17:e0262265. doi: 10.1371/journal.pone.0262265 35081135 PMC8791474

[ref129] O'Donnell K. McCormick S. P. Busman M. Proctor R. H. Ward T. J. Doehring G.. (2018). Toxigenic *Fusarium* species: identity and mycotoxicology revisited. Mycologia 110, 1058–1080. doi: 10.1080/00275514.2018.1519773 30481135

[ref131] Olt C. Faulkenberg K. D. Hsich E. M. (2021). The growing dilemma of legalized cannabis and heart transplantation. J. Heart Lung Transplant. 40, 863–871. doi: 10.1016/j.healun.2021.03.024 34006449 PMC10281819

[ref132] Ownley B. H. Gwinn K. D. Vega F. E. (2010). Endophytic fungal entomopathogens with activity against plant pathogens: ecology and evolution. BioControl 55, 113–128. doi: 10.1007/s10526-009-9241-x

[ref133] Palumbo J. D. O'Keeffe T. L. Mahoney N. E. (2007). Inhibition of ochratoxin a production and growth of *Aspergillus* species by phenolic antioxidant compounds. Mycopathologia 164, 241–248. doi: 10.1007/s11046-007-9057-0 17874203

[ref134] Pecoraro F. Giannini M. Beccari G. Covarelli L. Filippini G. Pisi A.. (2018). Comparative studies about fungal colonization and deoxynivalenol translocation in barley plants inoculated at the base with *Fusarium graminearum*, *Fusarium culmorum* and *Fusarium pseudograminearum*. AFSci. 27, 74–83. doi: 10.23986/afsci.67704

[ref135] Pérez-Moreno M. Pérez-Lloret P. González-Soriano J. Santos-Álvarez I. (2019). Cannabis resin in the region of Madrid: adulteration and contamination. Forensic Sci. Int. 298, 34–38. doi: 10.1016/j.forsciint.2019.02.049 30878463

[ref136] Perisetti A. Gajendran M. Dasari C. S. Bansal P. Aziz M. Inamdar S.. (2020). Cannabis hyperemesis syndrome: an update on the pathophysiology and management. Ann. Gastroenterol. 33, 571–578. doi: 10.20524/aog.2020.0528 33162734 PMC7599351

[ref138] Pitt J. I. (2002). Biology and ecology of toxigenic *Penicillium* species. Adv. Exp. Med. Biol. 504, 29–41. doi: 10.1007/978-1-4615-0629-4_4 11922096

[ref139] Pruyn S. A. Wang Q. Wu C. G. Taylor C. L. (2022). Quality standards in state programs permitting cannabis for medical uses. Cannabis Cannabinoid Res. 7, 728–735. doi: 10.1089/can.2021.0164 35363042 PMC11452082

[ref140] Punja Z. K. (2021a). Emerging diseases of *Cannabis sativa* and sustainable management. Pest Manag. Sci. 77, 3857–3870. doi: 10.1002/ps.6307 33527549 PMC8451794

[ref141] Punja Z. K. (2021b). First report of *Fusarium proliferatum* causing crown and stem rot, and pith necrosis, in cannabis (*Cannabis sativa* L., marijuana) plants. Can. J. Plant Pathol. 43, 236–255. doi: 10.1080/07060661.2020.1793222

[ref142] Punja Z. K. (2021c). Epidemiology of fusarium oxysporum causing root and crown rot of cannabis (*Cannabis sativa* L., marijuana) plants in commercial greenhouse production. Can. J. Plant Pathol. 43, 216–235. doi: 10.1080/07060661.2020.1788165

[ref143] Punja Z. K. (2021d). The diverse mycoflora present on dried cannabis (*Cannabis sativa* L., marijuana) inflorescences in commercial production. Can. J. Plant Pathol. 43, 88–100. doi: 10.1080/07060661.2020.1758959

[ref144] Punja Z. K. Collyer D. Scott C. Lung S. Holmes J. Sutton D. (2019). Pathogens and molds affecting production and quality of *Cannabis sativa* L. Front. Plant Sci. 10:1120. doi: 10.3389/fpls.2019.01120 31681341 PMC6811654

[ref145] Punja Z. K. Li N. Roberts A. J. (2021). The *Fusarium solani* species complex infecting cannabis (*Cannabis sativa* L., marijuana) plants and a first report of *fusarium (Cylindrocarpon) lichenicola* causing root and crown rot. Can. J. Plant Pathol. 43, 567–581. doi: 10.1080/07060661.2020.1866672

[ref146] Punja Z. K. Ni L. (2022). The bud rot pathogens infecting cannabis (*Cannabis sativa* L., marijuana) inflorescences: symptomology, species identification, pathogenicity and biological control. Can. J. Plant Pathol. 43, 827–854. doi: 10.1080/07060661.2021.1936650

[ref147] Punja Z. K. Ni L. Lung S. Buirs L. (2023). Total yeast and mold levels in high THC-containing cannabis (*Cannabis sativa* L.) inflorescences are influenced by genotype, environment, and pre-and post-harvest handling practices. Front. Microbiol. 14:1192035. doi: 10.3389/fmicb.2023.1192035 37383630 PMC10294073

[ref148] Punja Z. K. Scott C. S. (2023). Organically grown cannabis (*Cannabis sativa* L.) plants contain a diverse range of culturable epiphytic and endophytic fungi in inflorescences and stem tissues. Botany 101, 255–269. doi: 10.1139/cjb-2022-0116

[ref149] Rafei P. Englund A. Lorenzetti V. Elkholy H. Potenza M. N. Baldacchino A. M. (2023). Transcultural aspects of cannabis use: a descriptive overview of cannabis use across cultures. Curr. Addict. Rep. 10, 458–471. doi: 10.1007/s40429-023-00500-8

[ref150] Rakk D. Kukolya J. Škrbić B. D. Vágvölgyi C. Varga M. Szekeres A. (2023). Advantages of multiplexing ability of the orbitrap mass analyzer in the multi-mycotoxin analysis. Toxins 15:134. doi: 10.3390/toxins15020134 36828448 PMC9965799

[ref151] Ranjith A. Srilatha C. M. Lekshmi P. C. Rameshbabu N. (2021). Antiaflatoxigenic potential of essential oils of spices—a review. World Mycotoxin J. 14, 463–475. doi: 10.3920/WMJ2020.2636

[ref152] Raters M. Matissek R. (2008). Thermal stability of aflatoxin B1 and ochratoxin a. Mycotoxin Res. 24, 130–134. doi: 10.1007/BF03032339 23604747

[ref153] Reijula K. Tuomi T. (2003). Mycotoxins of aspergilli: exposure and health effects. Front. Biosci. 8, s232–s235. doi: 10.2741/978 12700107

[ref154] Remington T. L. Fuller F. Chiu I. (2015). Chronic necrotizing pulmonary aspergillosis in a patient with diabetes and marijuana use. CMAJ 87, 1305–1308. doi: 10.1503/cmaj.141412 PMC4646751 26100839

[ref155] Root K. S. Magzamen S. Sharp J. L. Reynolds S. J. Van Dyke M. Schaeffer J. W. (2020). Application of the environmental relative moldiness index in indoor marijuana grow operations. Ann. Work Expo. Health 64, 728–744. doi: 10.1093/annweh/wxaa071 32706020

[ref156] Ryan K. S. Bash J. C. Hanna C. B. Hedges J. C. Lo J. O. (2021). Effects of marijuana on reproductive health: preconception and gestational effects. Curr. Opin. Endocrinol. Diabetes Obes. 28, 558–565. doi: 10.1097/MED.0000000000000686 34709212 PMC8580253

[ref157] Ryan J. E. Noeder M. Burke C. Stubblefield S. C. Sulieman S. Miller E. G. (2019). Denying renal transplantation to an adolescent medical cannabis user: an ethical case study. Pediatr. Transplant. 23:e13467. doi: 10.1111/petr.13467 31124250 PMC6671627

[ref158] Salam A. P. Pozniak A. L. (2017). Disseminated aspergillosis in an HIV-positive cannabis user taking steroid treatment. Lancet Infect. Dis. 17:882. doi: 10.1016/S1473-3099(17)30438-3 28741551

[ref159] Samson R. A. Yilmaz N. Houbraken J. Spierenburg H. Seifert K. A. Peterson S. W.. (2011). Phylogeny and nomenclature of the genus *Talaromyces* and taxa accommodated in *Penicillium* subgenus *Biverticillium*. Stud. Mycol. 70, 159–183. doi: 10.3114/sim.2011.70.04 22308048 PMC3233910

[ref160] Sarma N. D. Waye A. ElSohl M. A. Brown P. N. Elzinga S. Johnson H. E.. (2020). Cannabis inflorescence for medical purposes: USP considerations for quality attributes. J. Nat. Prod. 83, 1334–1351. doi: 10.1021/acs.jnatprod.9b01200 32281793

[ref161] Scott C. Punja Z. K. (2023). Biological control of *Fusarium oxysporum* causing damping-off and Pythium myriotylum causing root and crown rot on cannabis (*Cannabis sativa* L.) plants. Can. J. Plant Pathol. 45, 238–252. doi: 10.1080/07060661.2023.2172082

[ref162] Shankar J. Tiwari S. Shishodia S. K. Gangwar M. Hoda S. Thakur R.. (2018). Molecular insights into development and virulence determinants of aspergilli: a proteomic perspective. Front. Cell. Infect. Microbiol. 8:180. doi: 10.3389/fcimb.2018.00180 29896454 PMC5986918

[ref163] Shapiro B. B. Hedrick R. Vanle B. C. Becker C. A. Nguyen C. Underhill D. M.. (2018). Cryptococcal meningitis in a daily cannabis smoker without evidence of immunodeficiency. BMJ Case Rep. 2018:bcr2017221435. doi: 10.1136/bcr-2017-221435 PMC5787011 29374632

[ref164] Slinski S. L. Zakharov F. Gordon T. R. (2015). The effect of resin and monoterpenes on spore germination and growth in *Fusarium circinatum*. Phytopathology 105, 119–125. doi: 10.1094/PHYTO-02-14-0027-R 25163010

[ref165] Smith H. Szarka D. Dixon E. Adedokun O. Munir M. Ricciardi M.. (2023). Emerging *Fusarium* spp. causing head blight on hemp in Kentucky. Plant Health Prog. 24, 132–134. doi: 10.1094/PHP-09-22-0089-SC

[ref166] Snigdha M. Hariprasad P. Venkateswaran G. (2015). Transport via xylem and accumulation of aflatoxin in seeds of groundnut plant. Chemosphere 119, 524–529. doi: 10.1016/j.chemosphere.2014.07.033 25112578

[ref167] Soliman S. S. M. Baldin C. Gu Y. Singh S. Gebremariam T. Swidergall M.. (2021). Mucoricin is a ricin-like toxin that is critical for the pathogenesis of mucormycosis. Nat Microbiol. 6, 313–326. doi: 10.1038/s41564-020-00837-0 33462434 PMC7914224

[ref168] Southall A (2022). What’s in New York’s illicit cannabis: germs, toxins and metals. Available at: https://www.nytimes.com/2022/12/01/nyregion/cannabis-bacteria-pesticides-illegal-dispensary.html (Accessed August 2, 2023).

[ref171] Steenwyk J. L. Mead M. E. Knowles S. L. Raja H. A. Roberts C. D. Bader O.. (2020). Variation among biosynthetic gene clusters, secondary metabolite profiles, and cards of virulence across *Aspergillus* species. Genetics 216, 481–497. doi: 10.1534/genetics.120.303549 32817009 PMC7536862

[ref172] Steenwyk J. L. Shen X. X. Lind A. L. Goldman G. H. Rokas A. (2019). A robust phylogenomic time tree for biotechnologically and medically important fungi in the genera *Aspergillus* and *Penicillium*. MBio 10, e00925–e01019. doi: 10.1128/mBio.00925-19 31289177 PMC6747717

[ref173] Stempfer M. Reinstadler V. Lang A. Oberacher H. (2021). Analysis of cannabis seizures by non-targeted liquid chromatography-tandem mass spectrometry. J. Pharm. Biomed. Anal. 205:114313. doi: 10.1016/j.jpba.2021.114313 34474231

[ref174] Stone T. Henkle J. Prakash V. (2019). Pulmonary mucormycosis associated with medical marijuana use. Respir. Med. Case. Rep. 26, 176–179. doi: 10.1016/j.rmcr.2019.01.008 30671341 PMC6330507

[ref175] Suga H. Hayashi M. Kushiro M. Miyano N. Inoue H. Nakajima K.. (2022). A novel medium for isolating two Japanese species in the *Fusarium graminearum* species complex and a dipstick DNA chromatography assay for species identification and trichothecene typing. J. Fungi 8:1048. doi: 10.3390/jof8101048 PMC9605519 36294613

[ref176] Sugui J. A. Pardo J. Chang Y. C. Zarember K. A. Nardone G. Galvez E. M.. (2007). Gliotoxin is a virulence factor of *Aspergillus fumigatus*: gliP deletion attenuates virulence in mice immunosuppressed with hydrocortisone. Eukaryot. Cell 6, 1562–1569. doi: 10.1128/EC.00141-07 17601876 PMC2043361

[ref177] Sweany R. R. Breunig M. Opoku J. Clay K. Spatafora J. W. Drott M. T.. (2022). Why do plant-pathogenic fungi produce mycotoxins? Potential roles for mycotoxins in the plant ecosystem. Phytopathology 112, 2044–2051. doi: 10.1094/PHYTO-02-22-0053-SYM 35502928

[ref178] Szyper-Kravitz M. Lang R. Manor Y. Lahav M. (2001). Early invasive pulmonary aspergillosis in a leukemia patient linked to *Aspergillus* contaminated marijuana smoking. Leuk. Lymphoma 42, 1433–1437. doi: 10.3109/10428190109097776 11911432

[ref179] Taghinasab M. Jabaji S. (2020). Cannabis microbiome and the role of endophytes in modulating the production of secondary metabolites: an overview. Microorganisms 8:355. doi: 10.3390/microorganisms8030355 32131457 PMC7143057

[ref180] Tapwal A. Nisha G. S. Gautam N. Kumar R. (2011). In vitro antifungal potency of plant extracts against five phytopathogens. Braz. Arch. Biol. Technol. 54, 1093–1098. doi: 10.1590/S1516-89132011000600003

[ref181] Trivedi P. Leach J. E. Tringe S. G. Sa T. Singh B. K. (2020). Plant-microbiome interactions: from community assembly to plant health. Nat. Rev. Microbiol. 18, 607–621. doi: 10.1038/s41579-020-0412-1 32788714

[ref182] Trullas J. -C. Bisbe J. Miro J. M. (2010). “Aspergillosis in drug addicts” in Aspergillosis: From Diagnosis to Prevention. ed. Pasqualotto A. C. (Berlin, DE: Springer Science+Business Media), 545–558.

[ref184] United States Department of Agriculture (USDA) (2020). Economic viability of industrial hemp in the United States: a review of state pilot programs. Available at: https://www.ers.usda.gov/webdocs/publications/95930/eib-217.pdf?v=4476.8 (Accessed August 3, 2023).

[ref185] United States Department of Agriculture-Economics, Statistics and Market Information System (USDA ESMIS) (2023). National hemp report. Available at: https://downloads.usda.library.cornell.edu/usda-esmis/files/gf06h2430/76538f824/w9506f61g/hempan23.pdf (Accessed May 1, 2023).

[ref186] United States Environmental Protection Agency (US EPA) (2023). Available at: https://www.epa.gov/pesticide-registration/pesticide-products-registered-use-hemp (Accessed August 2, 2023).

[ref187] United States Federal Grain Inspection Services (FGIS) (2023). FGIS approved mycotoxin rapid test kits. Available at: https://www.ams.usda.gov/sites/default/files/media/FGISApprovedMycotoxinRapidTestKits.pdf (Accessed July 19, 2023).

[ref188] United States Food and Drug Administration (FDA) (2010). Guidance for industry and FDA: advisory levels for deoxynivalenol (DON) in finished wheat products for human consumption and grains and grain by-products used for animal feed. Available at: https://www.fda.gov/regulatory-information/search-fda-guidance-documents/guidance-industry-and-fda-advisory-levels-deoxynivalenol-don-finished-wheat-products-human (Accessed August 6, 2023).

[ref189] United States National Fungus Collections and Fungal Database (NFCFD) (2023). Available at: https://data.nal.usda.gov/dataset/united-states-national-fungus-collections-fungus-host-dataset (Accessed August 8, 2023).

[ref190] United States Pharmacopeia (USP) (2019). General chapter <561> articles of botanical origin. Available at: https://hmc.usp.org/system/files/general-chapters/GC-Pdfs_2023/561%20Articles%20of%20Botanical%20Origin.pdf (Accessed May 1, 2023).

[ref191] Vanhove W. Cuypers E. Bonneure A. J. Gotink J. Stassen M. Tytgat J.. (2018). The health risks of Belgian illicit indoor cannabis plantations. J. Forensic Sci. 63, 1783–1789. doi: 10.1111/1556-4029.13788 29637553

[ref192] Varga J. Frisvad J. C. Samson R. (2009). A reappraisal of fungi producing aflatoxins. World Mycotoxin J. 2, 263–277. doi: 10.3920/WMJ2008.1094

[ref193] Veit M. (2023). Quality requirements for medicinal cannabis and respective products in the European Union—status quo. Planta Med. 89, 808–823. doi: 10.1055/a-1808-9708 35338476

[ref194] Wang G. S. Buttorff C. Wilks A. Schwam D. Tung G. Pacula R. L. (2021). Changes in emergency department encounters for vomiting after cannabis legalization in Colorado. JAMA Netw. Open 4:e2125063. doi: 10.1001/jamanetworkopen.2021.25063 34533572 PMC8449280

[ref195] Wang Y. Wang L. Liu F. Wang Q. Selvaraj J. N. Xing F.. (2016). Ochratoxin a producing fungi, biosynthetic pathway and regulatory mechanisms. Toxins 8:83. doi: 10.3390/toxins8030083 27007394 PMC4810228

[ref196] Wei G. Ning K. Zhang G. Yu H. Yang S. Dai F.. (2021). Compartment niche shapes the assembly and network of *Cannabis sativa*-associated microbiome. Front. Microbiol. 12:714993. doi: 10.3389/fmicb.2021.714993 34675893 PMC8524047

[ref197] Wilcox J. Pazdanska M. Milligan C. Chan D. MacDonald S. J. Donnelly C. (2020). Analysis of aflatoxins and ochratoxin a in cannabis and cannabis products by LC-fluorescence detection using cleanup with either multiantibody immunoaffinity columns or an automated system with in-line reusable immunoaffinity cartridges. J. AOAC Int. 103, 494–503. doi: 10.5740/jaoacint.19-0176 31558181

[ref198] Williams K. Buchert S. (2020). International regulation and control of hemp and cannabinoids. Cayman Chemical News and Announcements. Available at: https://www.caymanchem.com/news/international-regulation-and-control-of-hemp-and-cannabinoids (Accessed August 3, 2023).

[ref199] Xiong Y. Li W. Wen Q. Xu D. Ren J. Lin Q. (2022). Aptamer-engineered nanomaterials to aid in mycotoxin determination. Food Control 135:108661. doi: 10.1016/j.foodcont.2021.108661

[ref200] Yin Y. Cai M. Zhou X. Li Z. Zhang Y. (2016). Polyketides in *Aspergillus terreus*: biosynthesis pathway discovery and application. Appl. Microbiol. Biotechnol. 100, 7787–7798. doi: 10.1007/s00253-016-7733-z 27455860

[ref201] Yin G. Zhang Y. Pennerman K. K. Wu G. Hua S. S. T. Yu J.. (2017). Characterization of blue mold Penicillium species isolated from stored fruits using multiple highly conserved loci. J. Fungi 3:12. doi: 10.3390/jof3010012 PMC5715957 29371531

[ref202] Zaka M. Hashmi S. Siddiqui M. Rahman L. Mushtaq S. Ali H.. (2021). Callus-mediated biosynthesis of ag and ZnO nanoparticles using aqueous callus extract of *Cannabis sativa*: their cytotoxic potential and clinical potential against human pathogenic bacteria and fungi. Green Process Synth. 10, 569–584. doi: 10.1515/gps-2021-0057

[ref203] Zhao Z. Lou Y. Shui Y. Zhang J. Hu X. Zhang L.. (2021). Ochratoxigenic fungi in post-fermented tea and inhibitory activities of *Bacillus* spp. from post-fermented tea on ochratoxigenic fungi. Food Control 126:108050. doi: 10.1016/j.foodcont.2021.108050

[ref204] Zingales V. Fernández-Franzón M. Ruiz M. J. (2020). Sterigmatocystin: occurrence, toxicity and molecular mechanisms of action—a review. Food Chem. Toxicol. 146:111802. doi: 10.1016/j.fct.2020.111802 33035632

